# Processing the Chinese Reflexive “ziji”: Effects of Featural Constraints on Anaphor Resolution

**DOI:** 10.3389/fpsyg.2016.00284

**Published:** 2016-04-14

**Authors:** Xiao He, Elsi Kaiser

**Affiliations:** ^1^ProSearch Strategies, Inc.Los Angeles, CA, USA; ^2^Department of Linguistics, University of Southern CaliforniaLos Angeles, CA, USA

**Keywords:** sentence processing, reflexive pronouns, Chinese, self-paced reading, blocking effects, binding theory

## Abstract

We present three self-paced reading experiments that investigate the reflexive *ziji* “self” in Chinese—in particular, we tested whether and how person-feature-based blocking guides comprehenders' real-time processing and final interpretation of *ziji*. Prior work claims that in Chinese sentences like “John thought that {I/you/Bill} did not like ZIJI,” (i) the reflexive ziji can refer to the matrix subject John if the intervening subject is also a third person entity (e.g., Bill), but that (ii) an intervening first or second person pronoun blocks reference to the matrix subject, causing *ziji* to refer to the first or second person pronoun. However, native speakers' judgments regarding the accessibility of long-distance antecedents are rather unstable, and researchers also disagree on what the exact configurations are that allow blocking. In addition, many open questions persist regarding the real-time processing of reflexives more generally, in particular regarding the accessibility (or lack thereof) of structurally unlicensed antecedents. We conducted three self-paced reading studies where we recorded people's word-by-word reading times and also asked questions that probed their off-line interpretation of the reflexive *ziji*. People's answers to the off-line questions show that blocking is not absolute: Comprehenders do allow significant numbers of non-local choices in both the first and the second person blocking conditions, albeit in small numbers. At the same time, the reading time data, particularly those from Experiments 2 and 3, show that comprehenders use person feature cues to quickly filter out inaccessible long-distance referents. The difference between on-line and off-line patterns points to the possibility that the interpretation of *ziji* unfolds over time: it seems that initially, during real-time processing, person-feature cues weigh more heavily and constrain what antecedent candidates get considered, but that at some later point, other kinds of information are also integrated and perhaps outweigh the person-feature constraint, resulting in consideration of referents that were initially “blocked” due to the person-feature constraint. In sum, in addition to the structural constraints identified in prior work, person-featural cues also play a key role in regulating the on-line processing of reflexives in Chinese.

## Introduction

In real-time language comprehension, a major challenge faced by comprehenders is the need to resolve dependency relationships, where the interpretation of one linguistic element depends on another. One such case is the interpretation of reflexive pronouns (e.g., *himself/herself*), which is traditionally argued to depend on a set of structural constraints. In English, for example, reflexives are constrained by a set of rules termed Binding Principle A (Chomsky, 1981, 1986). According to this principle, a reflexive [*himself* in (1)] can only refer to a referent within the local clause (*Bill*) and not a referent outside the local clause (*John*).

(1) John_1_ said that Bill_2_ disliked himself_*1∕2_.

While structural constraints seem to adequately capture the patterning of reflexives in many contexts, their influence on the real-time processing of reflexives is the subject of an ongoing debate. Specifically, researchers disagree on whether or not structural information has an immediate effect on what referents are considered potential antecedents. Early work by Nicol and Swinney (1989) and Sturt (2003), among others, showed that comprehenders' consideration of potential antecedent candidates is immediately determined by structural constraints. More recent evidence in the same direction comes from Xiang et al. (2009), Dillon et al. (2013) and others. Hence, according to these studies, structurally-incompatible/inaccessible referents [such as *John* in (1)] do not cause interference during the processing of reflexives.

However, other studies found that comprehenders do not fully abide by structural rules, at least in the early stage of processing (e.g., Badecker and Straub, 2002; Runner et al., 2006; Kaiser et al., 2009; Clackson and Heyer, 2014). These findings suggest that initial consideration of possible antecedents can be influenced by featural properties of potential referents (which can act as retrieval cues), such as person, animacy, and number, and that comprehenders at least temporarily consider feature-compatible but structurally inappropriate referents before they eventually reach the correct interpretation. Thus, opinions diverge regarding the role of structural constraints in the real-time interpretation of reflexives.

This situation is complicated by the fact that reflexives in non-English languages are not necessarily constrained by the same principles that govern English reflexives (e.g., Kuno, 1972; Sells, 1987; Iida and Sells, 1988; Jayaseelan, 1999; Sohng, 2004, for crosslinguistic examples in Japanese, Malayalam, Korean, etc.). In particular, the phenomenon of long-distance reflexivization, where reflexives are bound by antecedents outside the local domain, has attracted considerable attention and poses challenges for the traditional definition of Binding Principle A^1^. Long-distance reflexives exist in many languages, including Chinese, Japanese, Korean and Icelandic. For example, the Chinese reflexive *ziji* can be long-distance bound in configurations such as (2a):

(2a) John_1_ juede    Bill_2_ bu     xihuan ziji_1∕2_.
       John_1_ thought Bill_2_ NEG like     SELF_1∕2_
       ‘John_1_ thought that Bill_2_  did not like  him_1_/himself_2_' [*ziji* =
       ambiguous]

Here, the reflexive *ziji* can refer to either the local subject Bill or the long-distance matrix subject John^2^. It has often been noted that, cross-linguistically, long-distance reflexives are subject to various language-specific constraints, such as the kinds of clause types that allow long-distance binding (e.g., infinitivals, subjunctive clauses, indicative clauses), the animacy of the antecedent, the type of verb in the matrix clause, and the person features of the referents in the sentences (see Huang, 2000, for an overview).

In this paper, we present three self-paced reading experiments on the processing of the Chinese long-distance reflexive *ziji* “self,” in order to enrich our understanding of the real-time processing of reflexives from a cross-linguistic perspective. Looking at *ziji* allows us to see what happens in a language where the accessibility of potential antecedents is governed by referents' person features: More specifically, while *ziji* could potentially refer to any subject-position referent (local or non-local), *the person feature* of intervening referents plays a key role in determining the accessibility of a long-distance referent.

### Long-distance reflexives in chinese: blocking effects

Here, we take a closer look at the Blocking Effects that have been claimed to guide the interpretation of long-distance reflexives in Chinese. Let us consider (2b). If the local subject is the first person pronoun *wo “* I” or the second person pronoun *ni* “you,” the widespread claim in the theoretical literature is that *ziji* is bound by this local subject (*wo*/*ni* “I/you”) and “blocked” from reaching the matrix/non-local subject (“John” in 2b) (see Xu, 1993; Pan, 1997, 2001; Huang and Liu, 2001). In contrast, if the local subject is a third person referent (“Bill” in 2a), *ziji* can refer to either the local subject or the matrix subject. Hence, there is an *asymmetric* Blocking Effect: An intervening first or second person pronoun blocks long-distance binding whereas a third person referent does not.

(2b) John_1_ juede   wo_2_/ni_2_ bu     xihuan ziji_2_.
       John   thought I/you    NEG like      SELF
       ‘John thought that I/you did not like myself/yourself'

Various theoretical analyses have been proposed for this Blocking Effect. One widely used syntactic strategy is to argue that apparent long-distance binding effects can be derived from local dependencies. For example, Tang (1989) (see also Cole et al., 1990; Cole and Sung, 1994; Cole and Wang, 1996, etc.) analyzed long-distance binding as involving a series of movements at the level of logical form such that each movement satisfies the requirement of local binding. Under this view, long-distance binding in Chinese underlyingly satisfies Chomsky's Binding Principle A.

In addition to syntactic accounts, semantic accounts have been proposed. The most prominent of these attributes blocking to a perspectival conflict, and is based on the “direct discourse complementation” analysis of Kuno (1972). According to Kuno, when a third-person pronoun in an embedded clause refers to the matrix subject who is the speaker/thinker of the embedded clause, the embedded clause can underlyingly be a direct speech event so that the third person pronoun in the surface form is directly derived from an underlying first person pronoun. A sentence like “John_*i*_ said he_*i*_ hated pancakes” is derived from the underlying form “John said, ‘I hate pancakes’.”

Building on this, Huang et al. (1984) argued that when *ziji* is in an indirect/reported speech event inside an embedded clause and used as a long-distance reflexive, the embedded clause is derived from a direct speech event. Consider (2a). When *ziji* refers to the matrix subject *John*, the underlying form of the sentence is represented of (2a′) where *ziji* is replaced with the first person *wo* in the direct quote. Here, the first person pronoun *wo* refers to the matrix subject *John*. Hence, a long-distance co-referential interpretation is established. On the other hand, if *ziji* refers to the local subject *Bill*, (2a) is derived from the underlying form in (2a″).

(2a′) John_1_ juede    Bill_2_ bu     xihuan wo_1_.
        John_1_ thought Bill_2_ NEG like      I_1_
        ‘John_1_ thought: “Bill_2_ doesn't like me.” '

(2a″) John_1_ juede    Bill_2_ bu     xihuan ziji_2_.
         John_1_ thought Bill_2_ NEG like     SELF_2_
         ‘John_1_ thought: “Bill_2_ doesn't like himself_2_.” '

Huang et al. (1984) argued that this approach explains the Blocking Effect. Consider (2b). Based on Huang et al., if *ziji* in (2b) is long-distance bound, the sentence is represented as (2b′), with the original *ziji* in (2b) being represented as the second occurrence of *wo* “I” in (2b′). This second instance of *wo* is intended to refer to the matrix subject *John*, but such a co-referential relationship is not allowed because it results in a conflict in perspectives: The two occurrences of *wo* in (2b′) refer to different referents: the first one refers to the external speaker of the sentence, and the second one to the matrix subject John. Hence, there is a conflict in perspectives. According to Huang et al. (1984), this perspectival conflict is the cause of blocking. In contrast, if *ziji* is locally bound, the underlying form is as in (2b″), and there is no perspectival conflict.

(2b′) ^*^ John_1_ juede   wo_2_ bu     xihuan wo_2_.
         John     thought I     NEG like     I
         ^*^‘John   thought:    “I(= external    speaker)    don't    like
         me(= John)”.'

(2b″) John_1_ juede    wo_2_ bu     xihuan ziji_2_.
         John   thought I      NEG like     SELF
         ‘John thought: “I do not like myself.”'

What about second person blocking? Huang et al. (1984) capture this in a similar way. In (2b), if the embedded subject is *ni* “you” and if *ziji* refers to the matrix subject (*ziji* = John), the underlying direct speech representation of (2b) would be (2c′) where *ziji* is replaced by first person *wo*. Note, however, that inside the direct speech, the second person pronoun *ni* refers to an addressee from the perspective of the *speaker of the entire sentence*, and not from the perspective of the matrix subject John—although, inside the direct speech, the first person pronoun *wo* is anchored to the matrix subject John (me = John). Hence, within the direct quote, we have two different perspectives, which cause a perspectival conflict. According to Huang et al., this again blocks long-distance binding.

(2c′) ^*^ John_1_ juede    ni_2_   bu     xihuan wo_1_.
         John     thought you NEG like      I
         ^*^‘John thought: “you(= addressee) don't like me(= John)”.'

(2c″) John_1_ juede    wo_2_ bu     xihuan ziji_2_.
         John   thought I      NEG like     SELF
         ‘John thought: “I do not like myself”.'

Building upon Huang et al. (1984), Huang and Liu (2001) analyzed *ziji* by using a combination of structural and semantic principles. They argued that (i) locally bound *ziji* is a true reflexive and is governed by Binding Principle A and that (ii) long-distance bound *ziji* is a logophor and not subject to Binding Theory.

In sum, these kinds of approaches attribute the Blocking Effects of intervening first and second person pronouns to a perspective conflict that stems from the embedded clause being underlyingly represented as direct speech. Both first and second person pronouns cause a perspective clash when realized in the embedded subject position—unlike third person referents—and thus first and second person referents trigger a Blocking Effect^3^.

However, this characterization of blocking is not universally agreed upon. Native speakers' judgments vary regarding the ability of intervening *third* person referents to block long-distance binding. For example, Tang (1989) and Pollard and Xue (1998) treat blocking as a symmetric process whereby a *difference* in person feature between a local referent and a long-distance referent suffices to induce blocking (regardless of the person feature of the intervening referent). Based on this view, the matrix subject *wo* (“I”) in (3) cannot antecede the reflexive *ziji*, even though the intervening referent is third person. In other words, some claim that Blocking Effects arise not just with first and second person pronouns, but instead occur in any context where the person features of the local and the long-distance referent are different.

(3) wo_1_ juede    Bill_2_ bu     xihuan ziji_?1∕2_.
      I      thought Bill  NEG like     SELF
      ‘I thought that Bill did not like SELF.'

In light of the divergent native speaker judgments, it would seem that a psycholinguistic approach could help clarify the situation. However, while there exists a large body of experimental work on English reflexives, experimental research on *ziji* has only recently become more frequent. The work that has been done has led to mixed results. For example, Dillon et al. (2009) showed that non-c-commanding subjects did not cause immediate interference in the real-time processing of *ziji*. Based on this finding, Dillon et al. argued that comprehenders only search for structurally compatible referents—c-commanding subject-position referents. Hence, structural constraints play an immediate role. In contrast, Chen and Vasishth (2011) and Jäger et al. (2015b), using self-paced reading and eye-tracking respectively, found that intervening feature-compatible (animate) non-c-commanding subjects caused reliable interference. (For recent work on interference effects and a cue-based retrieval mechanism in German and Swedish, see Jäger et al., 2015a). Based on these findings, they argued that consideration of potential antecedent candidates also relies on featural cues (e.g., animacy). In related work that also points to featural effects, Schumacher et al. (2011) conducted an ERP experiment which found that self-directed verbs exhibit different ERP responses with first- and third-person interveners than with second person interveners.

### Aims of the present work

The three self-paced reading experiments presented here aim to broaden our understanding of *ziji* by looking at whether and how person-feature-based blocking guides comprehenders' real-time processing and final interpretation of *ziji*.

We have three main aims: First, we want to test to what extent first person and second person interveners block access to long-distance subjects. Even before we turn to the debate regarding third person interveners, it is worth emphasizing that although it is often claimed that long-distance antecedents are not possible in the presence of an intervening first or second person pronoun, judgments seem to actually be rather murky. For example, in our experience, explicitly eliciting judgments from native speakers yields a mixed set of responses. Indeed, when we probed this in an off-line pilot study with 30 Mandarin speakers, we found that people would accept the supposedly impossible long-distance antecedent for *ziji* in a first-person blocking condition [like (2b)] 36.2% of the time. This seems like a rather high number for an interpretation that is supposed to be unavailable/ungrammatical. In order to be able to make progress on this issue, we feel that an experimental investigation of large groups of native speakers is an important step.

Second, given the debate on whether blocking is asymmetric, the present experiments are intended to test whether intervening third person referents block long-distance antecedents like their first and second person counterparts. Thus, in addition to our observations and off-line data which suggest that blocking by first and second person interveners may not be as absolute as some claim, there also exists a fundamental debate—both theoretical and empirical—about what exactly can act as a blocker.

Lastly, while previous experimental work on *ziji* focused primarily on the effect of structural constraints on non-c-commanding subjects, the current experiments examine the real-time effect of a different kind of constraint, namely person-feature cues. (Related work by Schumacher et al., 2011 on person features is discussed in more detail below.) We look at whether in real-time, person-feature cues can immediately reduce interference from blocked/inaccessible long-distance c-commanding subjects.

The three experiments presented in this paper investigate both the on-line processing and the final off-line interpretation of the reflexive *ziji* in the presence of potential first person, second person and third person interveners. Experiment 1 focuses on first person and third person interveners, whereas Experiment 2 tests second person and third person interveners. Furthermore, by changing the type of verb used in the matrix clause in Experiment 3, we test what happens when it is no longer possible to interpret the embedded clause as a direct speech act produced by the matrix subject.

## Experiment 1: first person blocking

Experiment 1 is a self-paced reading study that investigates the effects of intervening first-person pronouns on the real-time processing and off-line interpretation of the reflexive *ziji*. Specifically, we look at whether the presence of an intervening first person pronoun can fully block access to the long-distance matrix subject, as predicted by Blocking. If an intervening first person pronoun acts as an absolute blocker, the reflexive *ziji* should not trigger any consideration of the matrix subject—i.e., we should not see any sign of interference from the matrix subject, either in participants' on-line reading times or off-line interpretations. In addition, given the debate about the (a)symmetry of blocking, we also test whether a *difference* in person feature between a local referent and a long-distance referent suffices to induce blocking. In other words, can an intervening third person referent block access to a matrix subject with a different person feature, such as a first person subject? If yes, this would be evidence in favor of symmetric analyses of blocking and against asymmetric analyses.

### Methods

#### Participants

Twenty adult native speakers of Mainland Mandarin Chinese (graduate students at the University of Southern California at time of testing) took part in Experiment 1 in exchange for USD 10. All of them had normal or corrected-to-normal vision and reported no known learning disabilities or hearing impairments. All studies reported in this paper were reviewed and approved by the University of Southern California University Park Institutional Review Board, which is fully accredited by the Association for the Accreditation of Human Research Protection Programs (AAHRPP). Due to the nature of the experiments, the Institutional Review Board determined that written consent was not needed.

#### Materials

We used a 2 × 2 design by manipulating the form of the matrix subject and the embedded subject (first person pronoun vs. third person pronoun). Sample sentences of the four conditions are in (4).

(4) Sample sentences for the four conditions 
1st-1st 我告诉别人我觉得自己明年可以考进好大学。
            **wo** gaosu bieren **wo** juede ***ziji***     mingnian keyi kaojin
            **I**    tell     others  **I**    think **SELF** next year able get-in
            hao   daxue.
            good college
            “I told others that I thought SELF could get into a good
            college next year.”

1st-3rd 我告诉别人李四觉得自己明年可以考进好大学。
            **wo** gaosu bieren **Lisi** juede ***ziji***     mingnian  keyi kaojin
            **I**    tell      others **Lisi** think **SELF** next year able get-in
            hao   daxue.
            good college.
            “I told others that Lisi thought SELF could get into a good
             college next year.”

3rd-1st 张三告诉别人我觉得自己明年可以考进好大学。
            **Zhangsan** gaosu bieren **wo** juede ***ziji***     mingnian keyi
            **Zhangsan** tell      others **I**    think **SELF** next year able
            kaojin hao  daxue.
            get-in good college.
            “Zhangsan told others that I thought SELF could get into
            a good college next year.”

3rd-3rd 张三告诉别人李四觉得自己明年可以考进好大学。
            **Zhangsan** gaosu bieren **Lisi** juede ***ziji***     mingnian keyi
            **Zhangsan** tell      others **Lisi** think **SELF** next year able
            kaojin hao   daxue.
            get-in good college.
            “Zhangsan told others that Lisi thought SELF could get 
            into a good college next year.”

We created 32 target items, all of which contained 11 words^4^. (See Supplementary Materials for a full list of targets used in the experiments reported in this paper). The first and the fourth words were the matrix subject and the embedded subject, separated by a verb (*gaosu* “tell”) and an object (*bieren* “others”) both of which remained the same across all target items. The embedded subject was followed by a verb and then the reflexive *ziji*. In this study as well as the other studies reported in this paper, the verbs (and other lexical items) used in the embedded clauses were designed to be semantically neutral, i.e., to allow *ziji* to be interpreted as referring to either the matrix subject (e.g., Zhangsan) or the embedded subject (e.g., Lisi). [For work on the effects of self- vs. other-directed verbs on *ziji*, see Schumacher et al. (2011), He (2014) and others.]

Following the critical word *ziji* were five words (spillover region). This spillover region is important, because it is well known that in self-paced reading studies, effects many not be detectable until one, two or even three words after the critical word (e.g., Badecker and Straub, 2002, and many others). Our target items used *ziji* in the subject position of an embedded clause, because this allowed us to have a spillover region without a clause boundary *inside* the spillover region. (Clause and sentence boundaries are known to result in “wrap-up” slowdowns, e.g., Warren et al., 2009, which could potentially mask other effects. Indeed, we find signs of wrap-up slowdowns on the last word in our items, but this final word is not relevant for our analyses).

We employed a Latin Square design, resulting in four lists. Each participant saw 32 targets (8 per condition) and 72 fillers, described below. Each target item appeared once in each list but in a different condition in each list. (All experiments reported here used a Latin Square design.)

In addition to the 32 targets, 72 filler items were created. None of the fillers contained *ziji*. In this experiment, as in the other two experiments reported in this paper, the filler items were similar in length to the targets, and also contained multiple clauses (e.g., “Little An suggested that I go to a very renowned seafood restaurant by the seaside” and “Little Zhang heard from others that Little Liu's brother made Little Xiao very depressed”).

All targets and fillers were followed by a forced-choice question with two possible answer choices shown on the screen. Target questions probed participants' interpretations of *ziji*, as shown in (5). Because antecedent choice questions could not be used in the 1st-1st condition, we included a referent unmentioned in the sentence as one of the two antecedent choices (6). The forced-choice questions after fillers asked about referents mentioned in the filler items (e.g., “Who recommended a seafood restaurant?” (Little An/I), “Who was very depressed?” Little Xiao / Little Zhang). Positions (left vs. right) of the answer choices for the forced-choice questions were counterbalanced.

(5) Sample comprehension question:
     **Sentence: Zhangsan** gaosu bieren **Lisi** juede ***ziji*** mingnian
     keyi kaojin hao daxue.
     ‘*Zhangsan told others that Lisi thought SELF could get into a*
     *good college next year.'*
   
     **Comprehension**  Shui mingnian keyi kaojin hao daxue?
     **question:**             ‘*Who can get into a good college next year?'*
                                 (A) Zhangsan                             (B) Lisi

(6) Sample comprehension question for the 1st–1st condition:
     **Sentence: wo** gaosu bieren  **wo**  juede  ***ziji***  mingnian   keyi
     kaojin hao daxue.
     ‘*I told others that I thought SELF could get into a good college*
      *next year.'*
   
     **Comprehension**  Shui mingnian keyi kaojin hao daxue?
     **question:**            ‘*Who can get into a good college next year?'*
                                (A) Wangwu                              (B) I

#### Procedure

We used a moving-window word-by-word self-paced reading paradigm (Just et al., 1982; see also Badecker and Straub, 2002). Participants were tested individually on a laptop computer, using the *Linger* software (D. Rohde, MIT; Rohde, 2010). They first read the instructions and then proceeded to the practice items. The experimental trials started after the practice items. Participants read sentences word-by-word by pressing the spacebar. When a sentence was finished, a comprehension question with two answer choices appeared at the center of the screen. Participants responded by pressing the F key for the answer on the left side or the H key for the answer on the right side.

### Predictions

#### Antecedent choices

If the blocking effect of the first person pronoun is absolute, long-distance antecedents should be available in the 3rd-3rd Condition but crucially not in the 3rd-1st Condition due to the first person intervener. We should also keep in mind that researchers disagree about whether intervening third person referents can induce blocking: While some argue that only first and second person interveners lead to blocking, others claim that any person-feature mismatch between long-distance and local referents leads to blocking. Hence, antecedent choices data from the 1st-3rd Condition allow us to obtain a clearer picture of the status of third person interveners.

#### Reading times

Reading time slowdowns are taken to indicate competition or interference (e.g., Badecker and Straub, 2002). We follow Badecker and Straub (2002) in assuming that if a reflexive has two “candidate antecedents,” then additional processing is required to select a unique antecedent, and this increase in processing load is reflected in slower reading times. In other words, competition/interference results in a reading time slowdown, relative to a situation where only one antecedent is being considered. Thus, the 1st-1st Condition should be read rapidly as it has only one referent, the first person pronoun. The 3rd-3rd Condition, on the other hand, should exhibit slowdowns at the reflexive and/or beyond, due to the third person matrix subject competing with the third person embedded subject.

What about the 3rd-1st Condition? If the first person intervener immediately excludes the long-distance matrix subject from the set of possible antecedents, the matrix subject should not cause interference, and this condition should not be read more slowly than the 1st-1st Condition. Alternatively, if the first person pronoun is not an absolute blocker, interference reflected in reading time slowdowns should arise. Predictions for the 1st-3rd Condition are similar to the 3rd-1st Condition. If this condition exhibits blocking as some have argued, no interference should be expected from the first person matrix subject. Otherwise, this condition should also exhibit interference from the matrix subject as reflected in significant reading time slowdowns.

### Data analysis

We used participants' accuracy on the unambiguous filler comprehension questions to check whether they were attending to the task. Since all participants correctly answered at least 90% of the questions, all participants' data were included in subsequent trimming and analyses.

Reading times smaller than 100 ms or above 4000 ms were excluded first. Then, data points were log-transformed to reduce the non-normality of residuals. Afterwards, reading times more than 2.5 standard deviations away from the mean by word and by condition were removed, resulting in the exclusion of approximately 2.7% of data points. Statistical analyses were conducted in R (Baayen et al., 2008; R Core Team, 2015, see also Baayen, 2008). Data for each of the first 10 word positions in the target items were analyzed using linear-mixed effects regression implemented in the R package *lme4* (Bates et al., 2014). Main effects and interaction effects were computed with the R package *car* (Fox and Weisberg, 2011).

Unless otherwise mentioned, logistic mixed-effects regression implemented in *lme4* was used to analyze antecedent choices data due to their binary nature (see Jaeger, 2008, for discussion). In the analyses of antecedent choices, we excluded the data from the 1st-1st Condition because the sentences in this condition only contained the first person pronoun *wo* “I,” and one of the two options for the comprehension questions in this condition was a referent unmentioned in the sentence [see (6)]. Participants chose the unmentioned referent on 1.25% of trials in this condition, presumably by mistake.

To specify the random effects in each mixed-effects model, we started with fully crossed and fully specified random effects, testing whether the model could converge. If the model did not converge, we then reduced the random effects until the model reached convergence (see Jaeger at http://hlplab.wordpress.com). We then used likelihood ratio tests to test each random effect and removed those that did not contribute significantly to the model.

### Results

#### Antecedent choices

**Figure 1:**
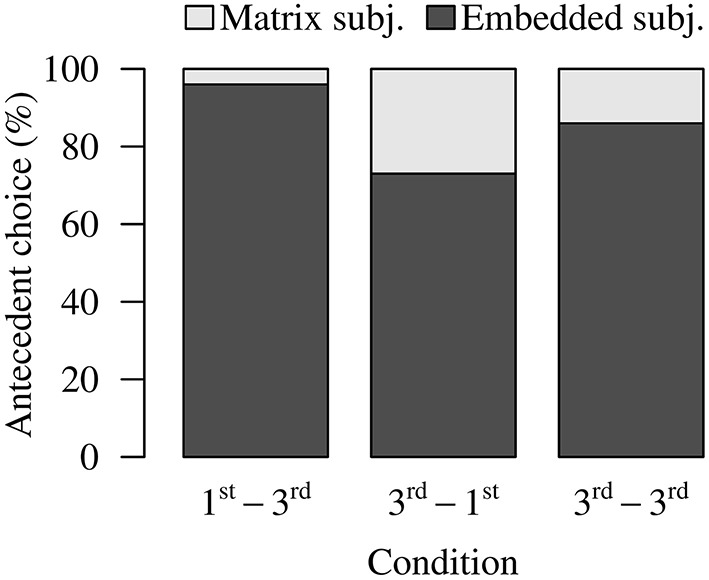
**Experiment 1 antecedent choice data**.

As Figure 1 shows, there was an overall preference for the local (embedded) subject in all conditions (1st-3rd: 95.92%; 3rd-1st: 73.12%; 3rd-3rd: 85.67%). This locality bias is expected based on earlier work (e.g., Chen et al., 2012; Dillon et al., 2014; Jäger et al., 2015b). Furthermore, we see a striking pattern in the 3rd-1st Condition: Although blocking predicts the 3rd-1st Condition to have the lowest rate of non-local choices, in this condition participants opted for the matrix subject and violated blocking on 26.88% of trials. The 3rd-3rd Condition, which—prior research agrees—permits non-local choices, actually had fewer non-local choices than the 3rd-1st Condition. Lastly, the 1st-3rd Condition numerically exhibited the fewest non-local antecedent choices (4.08%).

Antecedent choices were compared using logistic mixed-effects regressions. Participants chose the matrix subject significantly more in the 3rd-3rd and the 3rd-1st Conditions than in the 1st-3rd Condition (Table 1). Although the 3rd-1st Condition numerically produced more matrix subject choices than the 3rd-3rd Condition, this difference was not significant (Table 1). The higher-than-expected rate of matrix subject choices in the 3rd-1st Condition goes against the prediction of blocking. The low rate of matrix subject choices in the 1st-3rd Condition goes against the claim that third person interveners do not cause blocking.

**Table 1:** Experiment 1: Comparing the numbers of non-local antecedent choices in the 1st-3rd, the 3rd-1st, and the 3rd-3rd Conditions (“^*^”: *p* < 0.05; “.”: *p* < 0.1).

**Contrast**	**β**	***z***	**Pr(>|*z*|)**
3rd-1st vs. 1st-3rd	3.2087	2.732	<0.01^*^
3rd-3rd vs. 1st-3rd	3.1997	3.160	<0.005^*^
3rd-1st vs. 3rd-3rd	0.0090	0.016	1.0000

Lastly, we conducted Bonferroni-corrected one-sample t-tests to check whether the number of non-local, matrix subject choices in each condition was significantly above zero. (Here and elsewhere, we multiplied the *p*-values by the number of comparisons, instead of dividing the alpha level by the number of comparisons. These two options are mathematically equivalent.) The results showed that the amounts of non-local choices were significantly above zero in the 3rd-1st Condition [*t*_1(19)_ = 3.849, *p* < 0.010; *t*_2(31)_ = 6.294, *p* < 0.0001] and the 3rd-3rd Condition [*t*_1(19)_ = 5.511, *p* < 0.001; *t*_2(31)_ = 4.776, *p* < 0.0001]. For the 1st-3rd Condition, only the by-item test reached significance [*t*_1(19)_ = 1.926, *p* = 0.104; *t*_2(31)_ = 2.239, *p* = 0.4870]. Hence, the 3rd-1st Condition and the 3rd-3rd Condition and to a lesser extent the 1st-3rd Condition allow some amount of non-local choices.

#### Reading times

**Figure 2:**
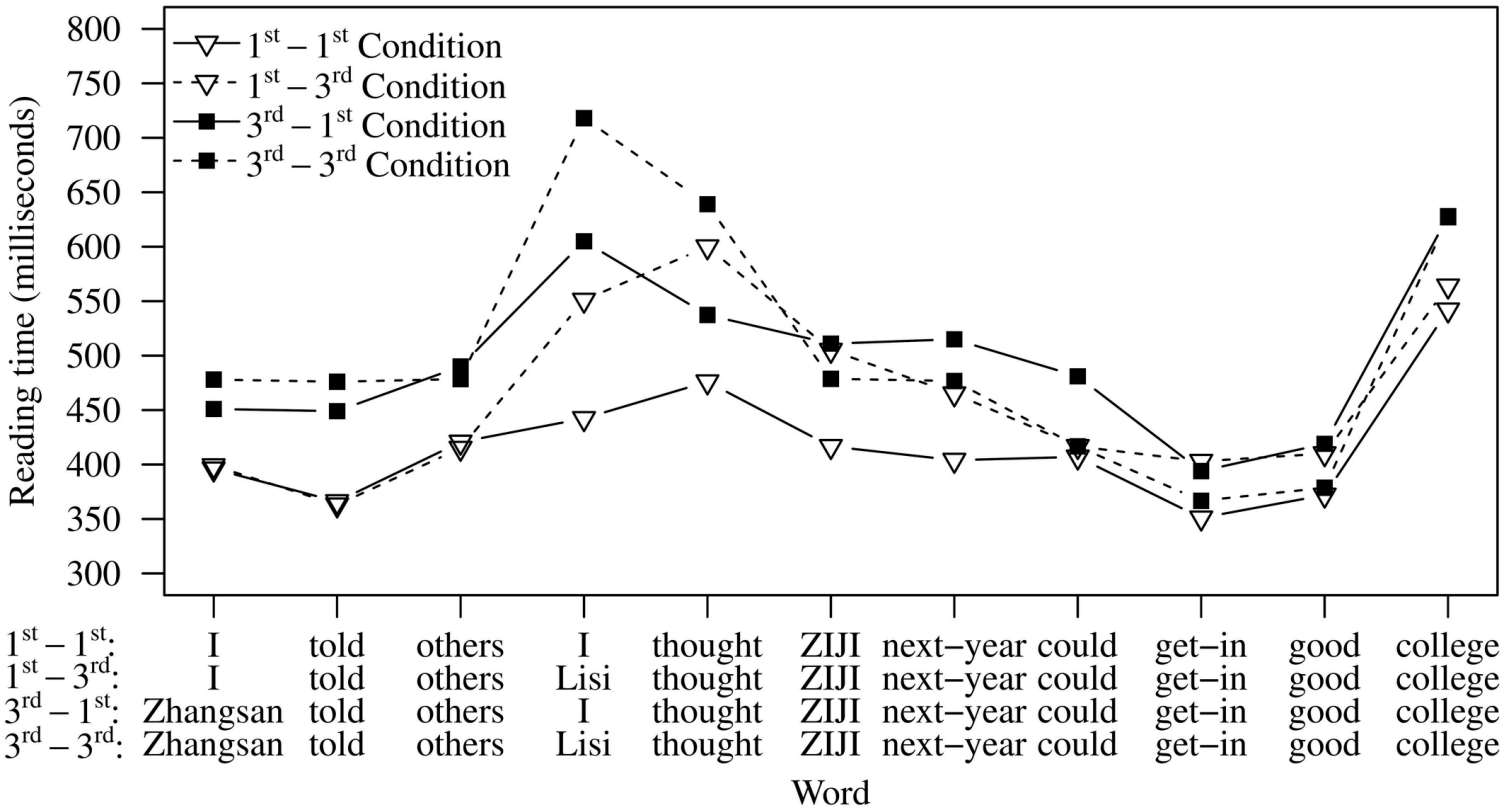
**Average reading times per word in Experiment 1**.

Reading time patterns are shown in Figure 2, and results of omnibus tests in Table 2. In the five word positions prior to the critical word *ziji*, significant effects of matrix subject were observed, suggesting that conditions with third person matrix subjects were read more slowly than those with first person matrix subjects. At the embedded subject and the following verb, significant effects of embedded subject emerged, indicating that conditions with third person embedded subjects were read more slowly. Existing work suggests that third person names are generally read more slowly than first and second person pronouns (Warren and Gibson, 2002), so these patterns are expected but are not central to the aims of this experiment, namely the processing of *ziji*.

**Table 2:** Experiment 1: Reading time results (“^*^”: *p* < 0.05; “.”: *p* < 0.1).

**Words**		**Main effects**						**Interaction**		
		**Matrix subject**			**Embedded subject**			**Matrix** ^*^ **Embedded**		
		**χ^2^**	***df***	***p***	**χ^2^**	***df***	***p***	**χ^2^**	***df***	***p***
1	I/Zhangsan	14.88	1	<0.001^*^	0.010	1	0.91	0.138	1	0.71
2	Told	86.53	1	<0.001^*^	0.000	1	1.00	0.366	1	0.55
3	Others	31.29	1	<0.001^*^	0.028	1	0.87	0.272	1	0.60
4	I/Lisi	36.34	1	<0.001^*^	18.40	1	<0.001^*^	1.928	1	0.17
5	thought	6.84	1	<0.010^*^	43.16	1	<0.001^*^	0.004	1	0.95
6	**ZIJI**	0.56	1	0.450	1.571	1	0.210	7.379	1	<0.01^*^
7	next year	6.37	1	<0.050^*^	3.178	1	0.075^·^	6.416	1	<0.05^*^
8	Could	0.053	1	0.820	0.014	1	0.906	2.836	1	0.09^·^
9	get in	0.065	1	0.799	1.152	1	0.283	7.656	1	<0.01^*^
10	Good	0.801	1	0.371	0.625	1	0.803	7.282	1	<0.01^*^

Starting from *ziji* (Word 6) and onward, a significant effect of matrix subject was observed at Word 7 (“next year”), but this effect was qualified by a matrix subject × embedded subject interaction. At *ziji* and several spillover words that followed, significant interaction effects were observed. To assess these interactions more closely, we compared the three two-referent conditions with the single-referent 1st-1st Condition. At word 7, all three double-referent conditions show significant slowdowns compared to the single-referent (1st-1st) condition (see Table 3). In fact, the 3rd-1st condition shows slowdowns relative to 1st-1st on *ziji* (word 6), word 7 as well as word 10. The 1st-3rd condition shows slowdowns relative to 1st-1st on *ziji* (word 6), word 7, and word 9.

**Table 3:** Experiment 1: Planned comparisons (“^*^”: *p* < 0.05; “.”: *p* < 0.1).

**Words**		**Contrasts**	**Results**			
			**β**	***SE***	***t***	**Pr(>|t|)**
6	ZIJI	1st-3rd vs. 1st-1st	0.1067	0.0381	2.804	0.0142^*^
		3rd-1st vs. 1st-1st	0.0936	0.0382	2.451	0.0384^*^
7	Next year	1st-3rd vs. 1st-1st	0.1305	0.0428	3.052	0.0064^*^
		3rd-1st vs. 1st-1st	0.1535	0.4293	3.576	0.0011^*^
		3rd-3rd vs. 1st-1st	0.1308	0.0428	3.057	0.0061^*^
9	Get in	1st-3rd vs. 1st-1st	0.0872	0.0321	2.714	0.0184^*^
10	Good	3rd-1st vs. 1st-1st	0.0721	0.0284	2.539	0.0303^*^

### Discussion

One of the goals of Experiment 1 was to look at whether the intervening first person pronoun constrains comprehenders' off-line interpretations of *ziji*. The **antecedent choices** in this experiment showed a higher-than-expected rate of matrix subject choices, indicating that blocking is not an absolute principle and that comprehenders sometimes do interpret *ziji* as referring to long-distance antecedents, even (or especially) in the presence of first person interveners. The current experiment also aimed to examine comprehenders' judgments of the 1st-3rd configuration. Researchers diverge regarding whether third person interveners can block access to person-feature mismatching long-distance referents (e.g., the 1st person matrix subject). Our data indicate the intervening third person referent in the 1st-3rd condition can “block” access to the long-distance subject in comprehenders' off-line judgments, in sense that we find less than 5% matrix-subject choices. This finding suggests that it is not accurate to analyze first person interveners as being “better” blockers than third person interveners, and supports previous research that treated third person referents as possible blockers as well (Tang, 1989; Pollard and Xue, 1998).

For the **reading time data**, the single-referent 1st-1st Condition and the double-referent 3rd-3rd Condition patterned as expected. The former was read fast, and the latter, in comparison, was read more slowly due to the two competing antecedents. This finding confirms the prediction that multiple accessible referents can cause competition, reflected in reading time slowdowns.

For the 3rd-1st Condition, if the intervening first person pronoun immediately constrains participants' consideration of antecedent candidates, the matrix subject should not be accessible and thus should not cause an interference (slowdown) effect. However, as we have seen, this condition gave rise to reading time slowdowns at *ziji* and in the spillover region, suggesting that the first person pronoun does not block the accessibility of the matrix subject in real-time and that the presence of two competing referents leads to an interference effect. This finding goes against theoretical claims which regard blocking as a categorical principle. However, it is in line with our off-line data which show that participants violated blocking on an unexpected high rate of trials in this condition.

Additionally, the 1st-3rd Condition, which produced the *fewest* non-local choices and hence exhibited a more stable blocking effect in the offline data, also showed reading time slowdowns. These slowdowns indicate that the “blocked” inaccessible matrix subject in sentences with this kind of configuration can still interfere with the local subject. The results from this condition are in line with existing work (e.g., Kaiser et al., 2009) that suggests comprehenders' off-line judgments do not always coincide with their real-time processing pattern. In our case, even though the off-line judgments suggest a stable blocking effect, in real-time, comprehenders can still briefly consider those “blocked” referents.

## Experiment 2: second person pronouns

In Experiment 1, we found a higher-than-expected rate of matrix-subject choices in the first person blocking condition, suggesting that first person blocking is not absolute and that comprehenders sometimes interpret *ziji* as referring the long-distance, matrix subject despite the presence of intervening first person pronouns. Additionally, the results also suggest that in terms of comprehenders' off-line judgments, the intervening third person subject can also serve as a blocker if the long-distance subject has a different person feature, such as first person in Experiment 1. To further examine the interpretation of *ziji* and the blocking effect, Experiment 2 looked at second person blocking. Based on existing theoretical work, we do not expect the first person pronoun and the second person pronoun to differ in their effectiveness as blockers. However, ERP work on Chinese by Schumacher et al. (2011) found that blocking configurations with first- vs. second-person pronouns triggered different brain responses. This brings up the question of whether first- and second-person pronouns could actually differ in their effectiveness as blockers. This idea receives preliminary (but indirect) support from Brunyé et al. (2009) work on English, which suggests that first- and second-person differ in their ability to induce perspective-taking (see also Ditman et al., 2010; Brunyé et al., 2011). This is especially interesting in light of claims by Huang and Liu (2001) and others that the Blocking effect in Chinese results from a perspective-taking process. In sum, there is (i) a need to better understand the strength of the Blocking effect, given the controversial judgments in this domain, and (ii) a need to better understand whether first- and second-person pronouns differ in their Blocking behavior. Answers to these questions can enrich our understanding of how reflexives are processed.

### Methods and data analysis

Twenty-eight adult native speakers of Mainland Mandarin Chinese (graduate students at the University of Southern California at time of testing) participated in exchange for USD 10. None of them took part in the previous experiment. All had normal or corrected-to-normal vision and reported no known learning disabilities or hearing impairments. The experimental design, materials, and procedure were identical to those used in Experiment 1, except that all the first person pronouns in the experimental items were replaced by second person pronouns (*ni* “you”). Like Experiment 1, this study also used a Latin Square design.

All participants were highly accurate on the comprehension questions for filler items (90% and above); thus, all participants' data were included in subsequent analyses. The trimming criteria were identical to Experiment 1, resulting in the exclusion of approximately 3.3% of data points. The same statistical methods used in the previous experiment were used here.

### Results

#### Antecedent choices

**Figure 3:**
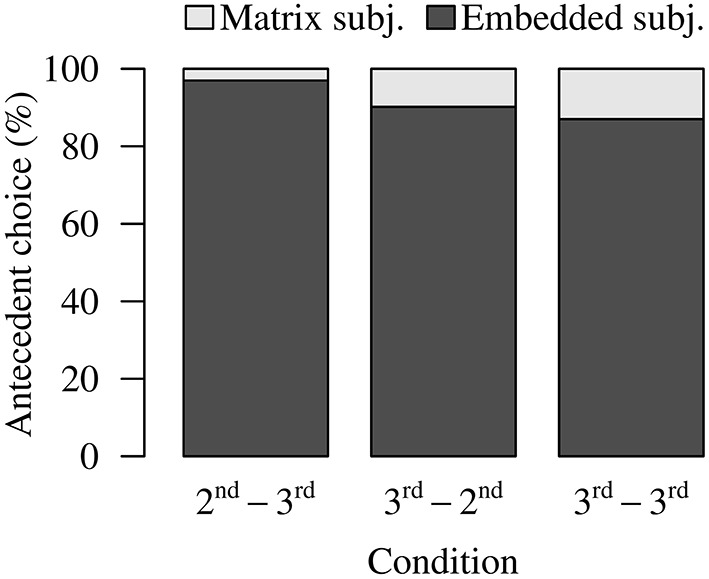
**Experiment 2 antecedent choice data**.

In line with the pattern observed in Experiment 1, there was an overall preference for local antecedent choices (Figure 3). In the 2nd-3rd, the 3rd-2nd, and the 3rd-3rd conditions, participants chose the embedded subject on 96.88%, 90.18%, and 87.05% of the trials, respectively (As in Experiment 1, we excluded the 2nd-2nd condition from this analysis because this condition only contained one referent, the second person pronoun *ni* “you”). We can also see that the 2nd-3rd Condition and the 3rd-3rd Condition in this experiment were numerically comparable to their counterparts in Experiment 1. However, long-distance choices were relatively rare in the 3rd-2nd Condition (9.82%), compared to the relatively high rate of long-distance choices in the 3rd-1st condition in Experiment 1 (26.88%). We conducted logistic mixed-effects regression to compare these three conditions. The results (Table 4) showed that the 3rd-3rd Condition produced significantly more long-distance choices than the 2nd-3rd Condition and the 3rd-2nd Condition. The 2nd-3rd Condition and the 3rd-2nd Condition did not differ significantly from each other.

**Table 4:** Experiment 2: Comparing the numbers of non-local antecedent choices in the 2nd-3rd, the 3rd-2nd, and the 3rd-3rd Conditions (“^*^”: *p* < 0.05; “.”: *p* < 0.1).

**Contrast**	**β**	***Z***	**Pr(>|*z*|)**
3rd-2nd vs. 2nd-3rd	0.1675	0.198	0.8433
3rd-3rd vs. 2nd-3rd	1.4281	3.199	<0.0100^*^
3rd-3rd vs. 3rd-2nd	1.8285	2.788	<0.0100^*^

Bonferroni-corrected by-subject and by-item one-sample t-tests were used to check whether the average number of non-local choices in each condition was significantly above zero. The results show that the number of non-local choices was significantly above zero in all three conditions [2nd-3rd: *t*_1(26)_ = 3.017, *p* < 0.010; *t*_2(31)_ = 3.950, *p* < 0.0010; 3rd-2nd: *t*_1(26)_ = 4.837, *p* < 0.001; *t*_2(31)_ = 6.428, *p* < 0.0001; 3rd-3rd: *t*_1(26)_ = 4.416, *p* < 0.001; *t*_2(31)_ = 2.234, *p* = 0.0492]. Hence, all three conditions allow non-local interpretations of *ziji* to a certain extent.

#### Comparing antecedent choices in experiments 1 and 2

In Experiment 1, with first person interveners, participants chose long-distance interpretations of *ziji* in the presence of first person blocking (3rd-1st Condition) on a considerable subset of trials (26.88%). In contrast, in Experiment 2, the 3rd-2nd Condition showed a numerically lower rate of matrix-subject choices (9.82%). Logistic mixed-effects regression was used to directly compare the antecedent choices in Experiment 1 (first person intervener) and Experiment 2 (second-person intervener). We found that the 3rd-1st Condition in Experiment 1 had significantly more matrix subject choices than the 3rd-2nd Condition in Experiment 2 (β = 2.0946, *z* = −2.444, *p* < 0.05). This difference suggests that relative to the first person pronoun, the second person pronoun constrains comprehenders' final interpretations of *ziji* more consistently. No significant differences were observed between the 1st-3rd Condition and the 2nd-3rd Condition (β = 0. 3152, *z* = 0.572, *p* = 1.000) or between the 3rd-3rd Condition from Experiment 2 and the 3rd-3rd Condition from Experiment 2 (β = 0.1637, *z* = 0.509, *p* = 1.000).

#### Reading times

**Figure 4:**
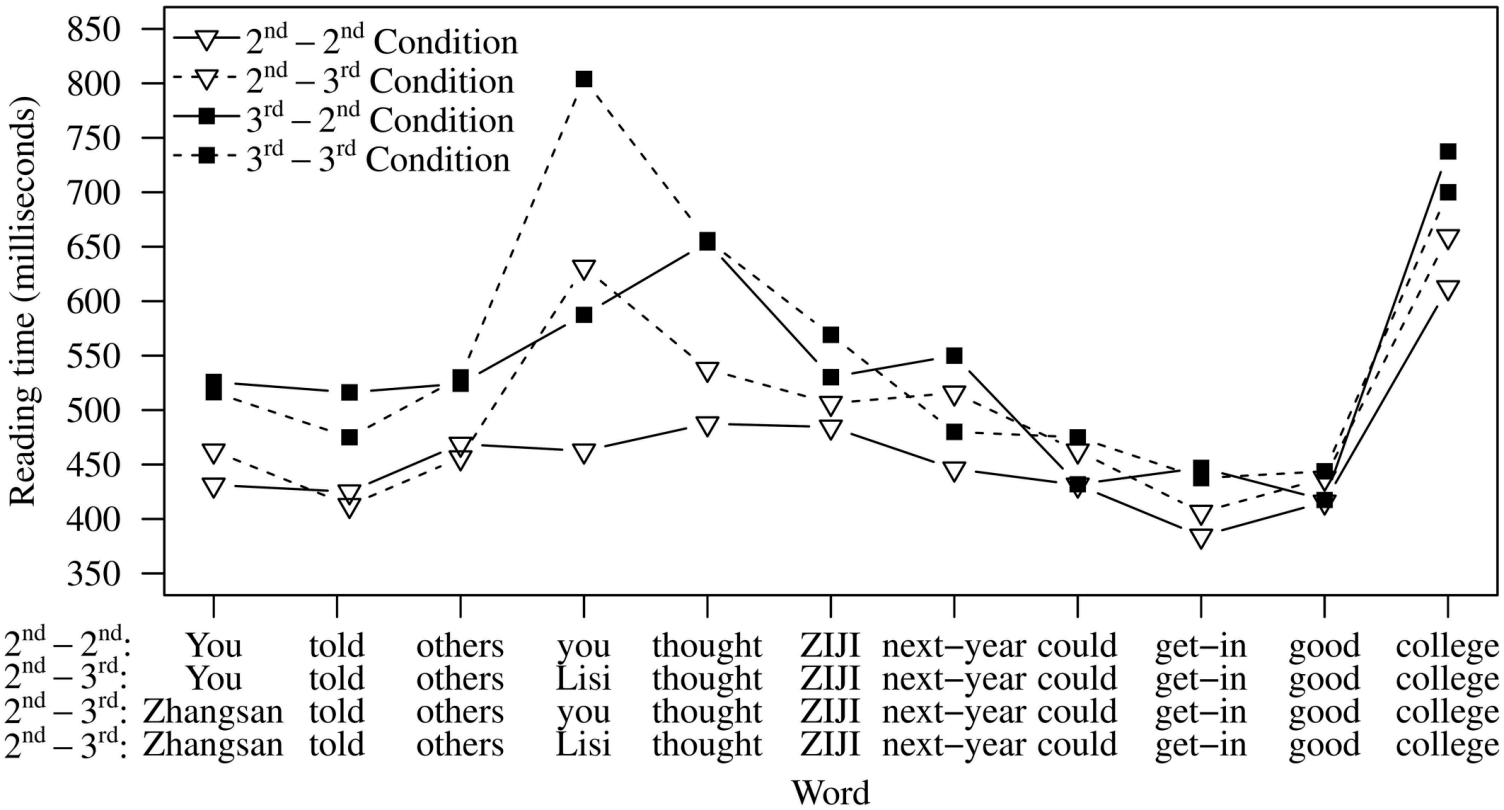
**Experiment 2 average reading time data**.

The reading time patterns for Experiment 2 are shown in Figure 4, and the results obtained from omnibus statistical tests are presented in Table 5. The five words preceding *ziji* show a pattern similar to Experiment 1. Significant main effects of matrix subject and embedded subject were observed, indicating that third person matrix and embedded subjects elicited longer reading times than their second person counterparts (Table 6). A significant interaction was also found at the embedded subject. A closer look at this interaction effect revealed that the 2nd-3rd Condition and the 3rd-3rd Condition were significantly slower than the single-referent 2nd-2nd Condition (2nd-3rd: β = 0.116, *z* = 2.301, *p* < 0.05; 3rd-3rd: β = 0.377, *z* = 6.90, *p* < 0.001) and that the slowdown in the 3rd-2nd Condition was marginally significant (β = 0.0703, *z* = 1.716, *p* = 0.087). As we already mentioned when discussing Experiment 1, which shows a very similar pattern at this point, these results are in line with existing work showing that third person names are generally read more slowly than reduced nouns such as first and second person pronouns (Warren and Gibson, 2002).

**Table 5:** Experiment 2: Reading time results (“^*^”: *p* < 0.05; “.”: *p* < 0.1).

**Words**		**Main effects**						**Interaction**		
		**Matrix subject**			**Embedded subject**			**Matrix** ^*^ **Embedded**		
		**χ^2^**	***df***	***p***	**χ^2^**	***df***	***p***	**χ^2^**	***df***	***p***
1	You/Zhangsan	19.38	1	<0.001^*^	1.122	1	0.270	0.496	1	0.481
2	Told	70.76	1	<0.001^*^	0.245	1	0.621	0.860	1	0.354
3	Others	20.52	1	<0.001^*^	0.004	1	0.951	0.626	1	0.429
4	you/Lisi	26.18	1	<0.001^*^	24.74	1	<0.001^*^	10.43	1	<0.001^*^
5	thought	20.72	1	<0.010^*^	10.82	1	<0.010^*^	0.054	1	0.817
6	**ZIJI**	0.938	1	0.333	8.932	1	<0.010^*^	0.635	1	0.425
7	next year	0.660	1	0.417	0.363	1	0.547	5.483	1	<0.050^*^
8	Could	0.023	1	0.374	0.791	1	0.374	0.100	1	0.752
9	get in	1.351	1	0.574	0.316	1	0.574	1.957	1	0.162
10	Good	2.722	1	0.099^·^	0.851	1	0.447	0.851	1	0.356

**Table 6:** **Target stimuli from Experiments 1 and 2**.

**1**	**2**	**3**	**4**	**5**	**6**	**7–11**
**Matrix subject**	**Verb**	**Object**	**Embedded subject**	**Verb**	**Reflexive**	**Spillover**
wo/Zhangsan “I/Zhangsan”	gaosu “told”	bieren “others”	I/Lisi	juede “thought”	ziji	mingnian keyikaojin hao daxue“next-year couldget-in goodcollege”
ni/Zhangsan “you/Zhangsan”			you/Lisi			

Exp 1: “{I/Zhangsan} told others that {I/Lisi} thought ZIJI could get into a good college next year.”

Exp 2: “{You/Zhangsan} told others that {you/Lisi} thought ZIJI could get into a good college next year.”

At the reflexive *ziji*, a significant main effect of embedded subject emerged, suggesting that the two conditions with third person embedded subjects (1st-3rd and 3rd-3rd Conditions) were read more slowly. The word immediately following *ziji* showed a significant interaction effect. Planned comparisons showed that the 2nd-3rd Condition and 3rd-2nd Condition were marginally slower than the 2nd-2nd Condition (2nd-3rd: β = 0.0691, *z* = 2.080, *p* = 0.096; 3rd-2nd: β = 0.0742, *z* = 2.23, *p* = 0.0672).

### Discussion

Building upon Experiment 1, Experiment 2 aimed to examine whether and how the intervening second person pronoun constrains comprehenders' interpretation of *ziji* both in real-time and off-line. The antecedent choice data provide additional insights into comprehenders' interpretations of the reflexive *ziji*. The results show that the second person blocking condition (3rd-2nd) exhibited a low rate of blocking violations. Direct comparisons of antecedent choice patterns between Experiments 1 and 2 confirm that the second person pronoun is indeed a more consistently effective blocker than the first person pronoun. The low rate of long-distance interpretations in the 2nd-3rd Condition, on the other hand, provides additional support for the claim that intervening third person referents can also cause blocking if the long-distance referent has a different person feature.

Using the reading time data, we aimed to examine whether the intervening second person can immediately constrain participants' consideration of potential antecedent candidates in real-time. If the effect of the second person intervener is similar to that of the first person intervener observed in Experiment 1, then an interference effect from the matrix subject should arise in the 3rd-2nd Condition. The results in Experiment 2 showed that the 3rd-2nd Condition was marginally slower than the single-referent 2nd-2nd Condition at the word immediately following *ziji*, but not at any of the subsequent spillover words. This contrasts with Experiment 1, which found significant slowdowns with first person interveners. This suggests that the second person can immediately determine what antecedent candidates get considered, i.e., that the matrix subject can be immediately excluded from consideration.

As a whole, the results from Experiment 2 show hints of the second person pronoun being a stronger blocker than the first person pronoun, given (i) the significantly *fewer* long-distance choices in the 3rd-2nd Condition compared to the 3rd-1st Condition in Experiment 1 and (ii) the absence of competition (i.e., absence of reading-time slowdowns) in the 3rd-2nd Condition.

In related ERP work, Schumacher et al. (2011) found differences between first person and second person interveners with self-directed verbs in constructions like “Wangwu asked me/you to examine myself/yourself.” They found a more pronounced early positivity with self-directed verbs with second person interveners than first or third person interveners. Self-directed verbs like “examine” tend to have objects that corefer with their subject/agents (Xi examined Xi). Schumacher et al. (2011) also note that sentences with the second person pronoun report a directive/imperative speech act whereas sentences with the first person pronoun report an assertive speech act. They suggest that, due to the imperative interpretation, the second person is higher on the person hierarchy than the first or the third person. Schumacher et al. (2011) also tested other-directed verbs and found no clear differences between first and second person interveners. Their results for self-directed verbs constitute the first published discussion of differences between first and second person interveners (to the best of our knowledge). However, our stimuli are different in a number of ways. Our target sentences used largely neutral verbs in the embedded clause (to allow *ziji* to refer to either the local or the matrix subject), and the matrix sentence used the verb “told others” (e.g., *Zhangsan told others that I/you/Lisi thought SELF could get into a good college next year*.) Thus, the addressee of “told” in our sentences is “the others,” and as a result an imperative interpretation is not possible, in contrast to Schumacher et al. (2011), who derive the different behavior of first and second person interveners from a hierarchy ranking related to the directive/imperative vs. assertive distinction.

As we will see below in Experiment 3, the apparent difference in the blocking strength of first and second person pronouns may in fact be a side effect having to do with participants' (mis)representing the embedded clauses in the test sentences as direct/quoted speech, rather than an intrinsic difference in the blocking behavior of these two forms.

## Experiment 3

Experiments 1 and 2 looked at the effects of first and second person blocking on the real-time processing and off-line interpretation of *ziji*. We saw that the first person pronoun did not seem to show a persistent blocking effect either off-line or in real-time. The second person pronoun, however, exhibited a more reliable blocking effect, significantly reducing interference from the matrix subject. This stronger blocking effect of second person interveners is not predicted by the majority of the existing literature on *ziji*—but see Schumacher et al.'s (2011)—and seems to point to a systematic difference in the blocking strength of the two pronouns.

However, let us take a careful look at the stimuli in Experiments 1 and 2, to see if there could be another reason for the asymmetry we observed. Target items had the sentence structure shown in Table 6: In both experiments, the main-clause verb (Word 2: *gaosu* “tell”) was a speech verb. Thus, the embedded clause (Words 4–11) following *gaosu bieren* “tell others” was indirect/reported speech.

For example, in (7), the embedded clause *wo juede ziji…* “I thought SELF…” is a reported speech event: Here, the person who uttered this sentence (the speaker) was reporting what Zhangsan said about the speaker's thoughts. Thus, the embedded subject *wo* “I” refers to the speaker of the entire sentence and not the matrix subject *Zhangsan*.

(7) Zhangsan gaosu bieren [wo juede    *ziji*     mingnian keyi
      Zhangsan tell     others [I    thought SELF next-year can 
      kaojin hao  daxue].
      get-in good college]
     *“Zhangsan told others that *[*I_speaker_ thought SELF could get*
     *into a good college next year*].”

(8) Zhangsan gaosu bieren: “wo juede     *ziji*    mingnian keyi
      Zhangsan tell     others: “I     thought SELF next-year can 
      kaojin hao  daxue.”
      get-in good college”
      *Zhangsan told others: “I_Zhangsan_ thought SELF could get into a*
      *good college next year*.”

However, we suggest that encountering a sentence like (7) may also activate, in people's minds, something similar to (8), which is direct/quoted speech. Crucially, if the embedded clause is direct/quoted speech spoken by Zhangsan, then *wo* “I” refers to Zhangsan and not the speaker of the sentence. (This idea differs from the earlier “transformation-based” approach of Kuno (1972) and Huang et al. (1984). We suggest that a sentence like (7) is, in some sense, ambiguous between reported speech and direct speech—or at least ambiguous enough that it at least partially activates a direct speech representation in participants' minds.) Let us now consider why we think that an example like (7) might partially activate a direct/quoted speech representation like (8).

First, Chinese lacks (overt) complementizers and hence a clause following a speech verb is (in terms of its lexical items) ambiguous between direct/quoted speech and indirect/reported speech. This is unlike English: Compare “*John said (that) I am tired”* with “*John said, ‘I am tired’*.” English does not use complementizers before direct speech, but optionally uses them before indirect speech. This probabilistic cue is entirely missing in Chinese. This ambiguity in Chinese may result in a sentence like (7) activating a direct speech representation (perhaps in parallel with an indirect speech representation or perhaps stochastically).

Second, the word-by-word self-paced reading paradigm may create the impression of potential “pauses” between words, which may make direct speech interpretations more likely. Given that the start of a direct/quoted speech event in spoken speech is typically characterized by a longer pause (Klewitz and Couper-Kuhlen, 1999), it could be that the boundaries between words created by the self-paced reading method led participants to activate a direct speech representation of the embedded clause. For example, it could be that the break between *bieren* “others” and *wo* “I” in (7) led participants to mentally represent (7) as (8) on some trials. (Like English, Chinese normally uses quotation marks to denote directed/quoted speech, but such cues—or the absence thereof—may be less salient in self-paced reading than normal reading which allows preview and regressions.)

In sum, we suggest that in Experiments 1 and 2, participants may have been partially activating direct speech representations, alongside indirect/reported speech representations. If participants are activating direct speech representations in addition to reported speech, this would lead precisely to the asymmetry between first and second person interveners that we found (i.e., first person pronouns seemingly acting as weaker blockers than second person pronouns):

In Experiment 1, with first person pronouns, under a direct speech representation, on blocking trials (3rd-1st Condition), the first person pronoun *wo* refers to the matrix subject [e.g., *wo* “I” = Zhangsan, as shown in (8)]. Then, if the reflexive *ziji* refers to *wo*, it also refers to the matrix subject [e.g., *Zhangsan* in (8)]. This might explain the apparent violations of blocking that occur on almost 30% of trials in the 3rd-1st condition of Experiment 1: The reflexive *ziji* only *seems* to skip the local subject in favor of the matrix subject: Actually, under a direct speech interpretation, *ziji* is bound by/refers to the local subject and thus also refers to the matrix subject, since local subject is coreferential with the matrix subject. So, according to this line of reasoning, the apparent long-distance interpretation is an *illusion* made possible by a direct speech interpretation, and *ziji* underlyingly refers to the local/embedded subject. Thus, if participants are partially activating direct speech representations alongside reported speech representations, on some proportion of the trials, the activation of the direct speech representation will presumably be sufficiently high to result in selection of the matrix subject.

In Experiment 2, with second person pronouns, the situation is different. In the blocking condition (3rd-2nd), whether or not comprehenders represent the embedded clause as direct speech, the second person pronoun *ni* “you” cannot refer to the matrix subject and can only refer to the addressee in both cases [see (9) and its direct speech counterpart (10)]. Thus, regardless of whether the embedded clause is interpreted as direct or indirect speech, the reflexive *ziji* in sentences with the second person pronoun *ni* “you” cannot use the same means to get to the matrix subject as in sentences with “I.” Therefore, the “escape hatch” that is possible with first person embedded subjects in direct speech is not possible with second person embedded subjects in direct speech.

(9) Zhangsan gaosu bieren [ni   juede     *ziji*     mingnian keyi
      Zhangsan tell     others [you thought SELF next-year can
      kaojin hao  daxue].
      get-in good college]
      “*Zhangsan told others that [youaddressee thought SELF could get*
      *into a good college next year].”*

(10) Zhangsan gaosu bieren: "ni juede    *ziji*     mingnian keyi
       Zhangsan tell      others: "ni thought SELF next-year can
       kaojin hao  daxue."
       get-in good college”
       “*Zhangsan told others: “You_addressee_ thought SELF could get*
       *into a good college next year*.”

This difference between first and second person pronouns fits with our results in Experiments 1 and 2, i.e., the finding that first person pronouns apparently fail to fully block reference to the matrix subject whereas second person pronouns are significantly stronger blockers. In sum, then, this line of reasoning explains the seemingly weaker blocking ability of first person pronouns as “illusory.” Under the direct-speech idea, the apparent weakness of first person pronouns as blockers stems from the fact that, with a speech verb in the matrix clause, first person pronouns can be coreferential with the matrix subject whereas second person pronouns cannot.

Experiment 3 aimed to investigate the validity of this idea. Instead of using speech verbs such as *gaosu* “to tell,” we used the serial verb structure *ting bieren shuo* “hear others say.” The use of the perception verb *ting* “hear” should eliminate the possibility that the embedded clause can be represented as the quoted direct speech of the matrix subject. Thus, Experiment 3 will allow us to see whether (i) the first person pronoun really is a weaker blocker than the second person pronoun, or whether (ii) the weakness of the first person pronoun as a blocker is actually due to direct speech representations.

### Methods

#### Participants

Forty-two adult native speakers of Mainland Mandarin Chinese from the Hunan Normal University in China participated in this experiment in exchange for 60 RMB (equivalent to 10 USD). All had normal or corrected-to-normal vision and reported no known learning disabilities or hearing impairments.

#### Design and stimuli

A 2 by 3 design was used. The first factor was pronoun type, with two levels—first person and second person. The second factor was referent combination, with three levels—pronoun-pronoun (or pro-pro) vs. pronoun-name (or pro-name) vs. name-pronoun (name-pro). This created a total of 6 conditions. The target sentence structure and examples of the 6 conditions are in Table 7.

**Table 7:** **Sentence structure for target items and sample target sentences in Experiment 3**.

**Condition**	**1**	**2**	**3**	**4**	**5**	**6**	**7**	**8**	**9–13**
**1st-1st**	**wo “**I”				**wo “**I”				
**1st-3rd**	**wo “**I”				**Lisi**				
**3rd-1st**	**Zhangsan**	ting “hear”	bieren “others”	shuo “say”	**wo “**I”	keyi “can”	BA	**ZIJI** “SELF”	de chengji rang bieren kan
**2nd-2nd**	**ni “**you”				**ni “**you”				“DE grade let others see”
**2nd-3rd**	**ni “**you”				**Lisi**				
**3rd-2nd**	**Zhangsan**				**ni “**you”				

**1st-1st: “I** heard others say **I** could give **ZIJI**'s score to others to look at.”

**1st-3rd: “*I*** heard others say **Lisi** could give **ZIJI**'s score to others to look at.”

**3rd-1st:** “**Zhangsan** heard others say **I** could give **ZIJI**'s score to others to look at.”

**2nd-2nd: “You** heard others say **you** could give **ZIJI**'s score to others to look at.”

**2nd-3rd: “You** heard others say **Lisi** could give **ZIJI**'s score to others to look at.”

**3rd-2nd: “Zhangsan** heard others say **you** could give **ZIJI**'s score to others to look at.”

A total of 42 target items were created, each with 13 words (Table 7). The first and the fifth words were the matrix subject and the embedded subject, respectively. The two subjects were separated by a serial verb—*ting biren shuo* “hear others say”^5^—that was constant across all target items. The reflexive was the eighth word and was in the possessive NP position (e.g., *ziji de chengji* “SELF's grade”). Finally, *ziji* was followed by five spillover words (Words 9–13). (The grammatical role of *ziji* in Experiment 3 is different from Experiments 1 and 2. This was necessitated by the change of verb from “tell others” to “hear others say,” because we wanted to ensure that the sentences were felicitous and that *ziji* could, in principle, be interpreted as referring to either the matrix or the embedded subject.)

Crucially, the use of the perception verb *ting* “hear” should eliminate the possibility of interpreting the embedded clause (Words 5-13) as the quoted direct speech of the matrix subject. In (11) for example, the embedded clause *wo keyi…* “I could…” cannot be the quoted speech of the matrix subject *Zhangsan*, and can only be what *Zhangsan* heard. Hence, the first person pronoun *wo* “I” refers to the person who uttered the entire sentence and cannot refer to the matrix subject (unlike Experiment 1).

(11) **Zhangsan** ting    bieren shuo [wo keyi    ba    *ziji*   de
       **Zhangsan** heard others say   [**I**    could  BA   **ZIJI** DE 
       chengji gei bieren kan.
       grade   let others see].
       ‘Zhangsan heard others say [I could let others see SELF's
       grade].‘

Like Experiments 1 and 2, this study also used a Latin Square design. Experiment 3 had six lists, due to its 2 by 3 design. Each participant saw 42 targets (seven per condition), and 68 fillers. The fillers were similar to those in Experiments 1 and 2 (see Section Materials). Similar to the previous two experiments, all items were followed by a forced-choice question. Left-vs.-right positions of the answer choices were counterbalanced.

#### Procedure

The experimental procedure in this experiment was identical to those in Experiments 1 and 2.

### Predictions

#### Antecedent choices

If the intervening first person pronoun indeed has a weaker blocking effect than the second person pronoun, we should observe a relatively high rate of blocking violations—i.e., matrix subject choices—in the conditions with first person interveners (3rd-1st) when compared to the conditions with second person interveners (3rd-2nd). Alternatively, if the weakness of the first person pronoun as a blocker (as in Experiment 1) is an illusion due to the “escape hatch” provided by direct speech representations which are ruled out in Experiment 3, then we expect the rate of blocking violations in conditions with first and second person interveners to now be comparable (a low number of blocking violations in both conditions).

#### Reading times

If the first person pronoun has a weaker blocking effect than the second person pronoun, then in conditions with first person interveners, we should see reading time slowdowns from *ziji* and onwards as a result of competitions between the matrix subject and the embedded subject. In particular, the reading times in the first person blocking condition should be slower compared to those in the second person blocking condition, if the first person pronoun is weaker blocker (i.e., allows more competition from the matrix subject) than the second person pronoun. On the other hand, if the blocking violations in Experiment 1 (first person interveners) were actually “illusions” due to direct speech, then in Experiment 3, we should not observe competitions between the blocked matrix subject and the local subject.

### Data analysis

All participants were highly accurate on the comprehension questions for filler items (90% and above); thus, all data were included in subsequent analyses. The trimming criteria were identical to those used in Experiments 1 and 2 and resulted in the exclusion of 2.59% of data points. The same statistical methods were used to analyze data in the present experiment. The reading time data for the first 12 words of the target items were analyzed.

### Results and discussion

In this section, we present the results of Experiment 3 and discuss them briefly. We postpone an in-depth discussion of Experiment 3 until the General Discussion, because the full import of this third study is best appreciated when it is compared to the results of Experiments 1 and 2.

#### Antecedent choices

**Figure 5:**
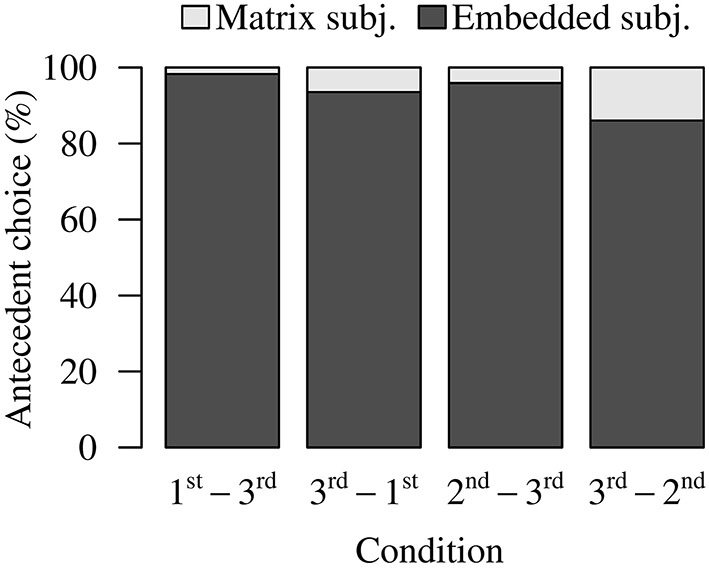
**Experiment 3 antecedent choice data**.

As shown in Figure 5, matrix subject choices were numerically relatively rare (1st-3rd: 1.70%; 3rd-1st: 6.46%; 2nd-3rd: 4.08%; 3rd-2nd: 13.95%). However, the first person blocking condition (3rd-1st) and the second person blocking condition (3rd-2nd) had somewhat higher percentages of matrix subject choices than the other conditions. Surprisingly, the 3rd-2nd Condition actually had numerically the *highest* rate of matrix subject choices. (As in the preceding studies, we excluded the data from the two single-referent conditions, 2nd-2nd and 1st-1st, from analysis the antecedent-choice analyses.)

Logistic mixed-effects regression was used to test the effects of pronoun type and referent combination on antecedent choices. The results showed significant main effects of pronoun type [χ^2^ = 13.069, *df* = 1, *p* < 0.001] and referent combination [χ^2^ = 21.071, *df* = 1, *p* < 0.001] but no interaction [χ^2^ = 0.0349, *df* = 1, *p* = 0.852]. Hence, conditions with second person pronouns were more likely to elicit matrix subject choices than conditions with first person pronouns, and the two blocking configurations were more likely to produce matrix subject choices than the other two conditions.

Although the omnibus test reported above did not yield a significant interaction, a set of four planned comparisons was carried out. The results (Table 8) showed that the 3rd-1st Condition elicited more matrix subject choices than the 1st-3rd Condition, and that the 3rd-2nd Condition elicited more matrix subject choices than the 2nd-3rd Condition. In addition, the 3rd-1st Condition actually had *fewer* matrix subject choices than the 3rd-2nd Condition. This result is different from what we saw in Experiments 1 and 2 where the opposite pattern was observed. That is, the 3rd-1st Condition in Experiment 1 led to significantly more matrix subject choices than the 3rd-2nd Condition in Experiment 2. The finding that in Experiment 3 (when direct speech interpretations are blocked), the 3rd-1st Condition resulted in *fewer* matrix subject choices than the 3rd-2nd Condition clearly argues against the idea that the first person is an inherently weaker blocker than the second person.

**Table 8:** Experiment 3: Planned comparisons for antecedent choice data (“^*^”: *p* < 0.05; “.”: *p* < 0.1).

**Contrast**	**β**	***z***	**Pr(>|*z*|)**
3rd-1st vs. 1st-3rd	2.0751	2.975	0.0107^*^
3rd-2st vs. 2nd-3rd	2.4489	4.618	<0.001^*^
3rd-1st vs. 3rd-2nd	−1.2112	−3.604	0.0012^*^
1st-3rd vs. 2nd-3rd	−0.8374	−1.051	0.6672

As with the first two experiments, we used Bonferroni-corrected one-sample *t*-tests to check whether the number of non-local, matrix subject choices in each condition was significantly above zero. The results showed that the numbers of matrix subject choices in the two blocking conditions were significantly above zero, and were marginally above zero in the 1st-3rd condition and significantly above zero in the 2nd-3rd condition [1st-3rd: *t*_1(41)_ = 2.354, *p* = 0.094; *t*_2(41)_ = 3.950, *p* = 0.094; 3rd-1st: *t*_1(41)_ = 2.953, *p* = 0.021; *t*_2(41)_ = 6.428, *p* < 0.001; 2nd-3rd: *t*_1(41)_ = 3.106, *p* = 0.014; *t*_2(41)_ = 3.950, *p* = 0.007; 3rd-2nd: *t*_1(41)_ = 3.683, *p* = 0.003; *t*_2(41)_ = 6.428, *p* < 0.001].

In sum, we find that the 3rd-1st and the 3rd-2nd Conditions, which are often regarded as the prototypical blocking conditions, allow rates of blocking violations (matrix subject choices) that are significantly higher than 0, and in fact higher than the 1st-3rd and 2nd-3rd Conditions respectively. This indicates that blocking is not a strict, categorical phenomenon. Furthermore, we find no evidence that first person pronouns are weaker blockers than second person pronouns. In fact, in this study, the 3rd-2nd Condition results in more matrix subject choices than the 3rd-1st condition. This suggests that the high rate of matrix subject choices in Experiment 1 may indeed have been due to participants activating direct speech representations, which function as an “escape hatch” to allow *ziji* to refer to the matrix subject in the presence of an intervening first-person subject.

#### Reading times

**Figure 6:**
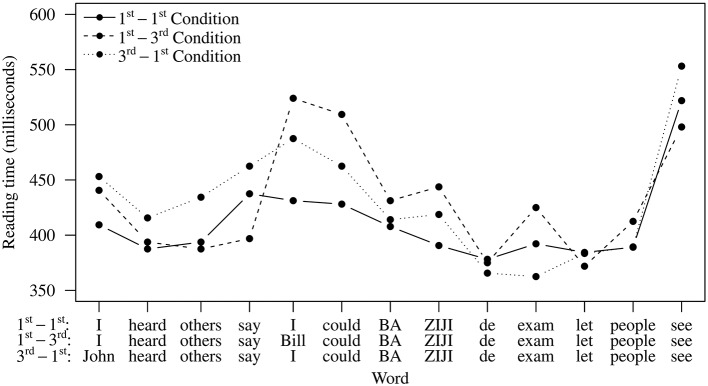
**Reading times for conditions with the first person pronoun in Experiment 3**.

**Figure 7:**
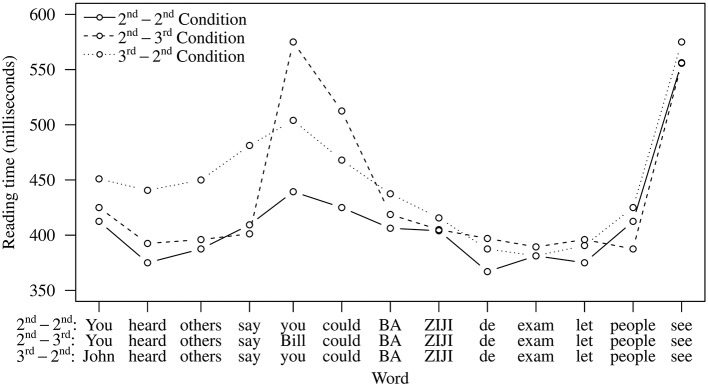
**Reading times for conditions with the second person pronoun in Experiment 3**.

The reading time patterns are presented in Figure 6 (conditions with first person pronouns) and Figure 7 (conditions with second person pronouns). Linear mixed-effects regression was used to analyze log-transformed reading time data.

At the 7 words prior to *ziji*, a persistent effect of referent combination was observed (Table 9), reflecting an increased processing effort involved in reading third person names (compared to first and second person pronouns). This pattern is similar to what we observed in Experiments 1 and 2 and is also expected based on existing research (Warren and Gibson, 2002). From *ziji* and onwards, marginally significant effects of referent combination were observed at Words 9 and 10. However, planned comparisons did not yield any significant differences at these positions. A significant referent combination × pronoun type interaction was found at word 12, but planned comparisons did not reveal any significant contrasts at this position.

**Table 9:** Experiment 2: Reading time results (“^*^”: *p* < 0.05; “.”: *p* < 0.1).

**Words**		**Main effect**						**Interaction**		
		**Referent combination**			**Pronoun type**					
		**χ^2^**	***df***	***p***	**χ^2^**	***df***	***p***	**χ^2^**	***df***	***p***
1	I/You/Zhangsan	13.71	2	<0.010^*^	1.136	1	0.287	1.419	2	0.492
2	Heard	81.29	2	<0.001^*^	1.908	1	0.167	1.844	2	0.398
3	Others	45.42	2	<0.001^*^	0.367	1	0.545	0.043	2	0.979
4	Say	36.12	2	<0.001^*^	0.010	1	0.921	2.916	2	0.233
5	I/you/Lisi	30.43	2	<0.001^*^	3.275	1	0.070.	1.300	2	0.522
6	Could	30.63	2	<0.001^*^	0.605	1	0.825	0.605	2	0.739
7	BA	16.31	2	<0.001^*^	0.775	1	0.379	3.772	2	0.152
8	**ZIJI**	3.801	2	0.150	0.000	1	0.995	3.602	2	0.165
9	DE	4.951	2	0.084.	2.951	1	0.086	1.276	2	0.528
10	Exam	5.937	2	0.051.	0.006	1	0.940	1.685	2	0.431
11	Let	0.189	2	0.910	0.761	1	0.383	3.633	2	0.393
12	People	1.248	2	0.546	3.917	1	0.048	8.371	2	0.015^*^

In sum, we find no clear evidence for reading-time slowdowns (i.e., competition between multiple referents) after *ziji*. This suggests that in this experiment, when direct speech representations are not possible, participants are not considering the matrix subject as a potential referent—or not sufficiently for it to result in a reading-time slowdown—for the reflexive *ziji*. In the General Discussion, we take a closer look at how these results relate to the outcomes of Experiments 1 and 2, and what the implications of these comparisons are.

## General discussion

The experiments reported here tested whether and how person-feature-based blocking guides comprehenders' real-time processing and final interpretation of the Chinese reflexive *ziji* “self.” Our work was motivated by three main aims. First, we wanted to test experimentally to what extent native speakers' judgments fit with the view often presented in theoretical work that first person and second person interveners block access to long-distance subjects. Second, there is debate in existing work concerning the configurations that can result in blocking—in particular, whether blocking only occurs with intervening first and second person pronouns, or whether it can also occur with third person pronouns as long as there exists a mismatch in the featural make-up of the matrix subject and the embedded subject. We tested whether intervening third person referents block long-distance antecedents like their first and second person counterparts. Third, we complement prior on-line work by testing whether person-feature cues can immediately reduce interference from blocked / inaccessible long-distance c-commanding subjects.

### Absence of absolute blocking effects, and potential asymmetries between first and second person pronouns

Regarding the first and third questions mentioned above, Experiment 1 found that first person interveners in the purported blocking condition (3rd-1st) resulted in a higher-than-expected rate of matrix subject choices, as well as reading-time slowdowns. This suggests that when comprehenders encounter sentences with third person matrix subjects, first person embedded subjects, and a reflexive *ziji* in the embedded clause (3rd-1st), both the embedded and matrix subject compete as potential antecedents for the reflexive. This argues against claims that blocking is categorical, since under that view, we should see no matrix subject choices and no slowdowns. Interestingly, Experiment 2 found that second person interveners in the purported blocking condition (3rd-2nd) exhibited a low rate of matrix subject choices and only short-lived, marginal reading-time slowdowns.

In light of these results, one might be tempted to conclude that second person pronouns are stronger blockers. Such a conclusion might in fact be expected, in light of earlier claims that blocking in Chinese is related to perspective taking (Huang and Liu, 2001). Let us combine this idea with other work on perspective in cognitive psychology which found that in English (at least in some contexts), the second person induces stronger perspective-taking than the first person (Brunyé et al., 2009; Ditman et al., 2010, see also Brunyé et al., 2011). If this stronger perspective-taking effect with the second person pronoun also holds in Chinese and if blocking is indeed related to perspective taking, then we may expect to see a stronger blocking effect with the second person pronoun. However, as we will see below, Experiment 3 shows that this conclusion is too hasty, because the use of a verb of saying as the embedding verb in Experiments 1 and 2 allows first-person pronouns an “escape hatch” that seems to be boosting the rate of matrix subject choices without violating blocking.

### Evidence for blocking in asymmetrical environments, even without first or second person blockers

Regarding the second question above, namely whether blocking (even if it is not absolute) only occurs when the embedded subject (the blocker) is first or second person or whether blocking phenomena can also occur in configurations where the two subjects have different person features (e.g., 1st-3rd or 2nd-3rd, so the blocker is third person), our results argue for the second view. In all three experiments, an intervening third person referent in the 1st-3rd and 2nd-3rd conditions can block access to the long-distance subject in off-line judgments (i.e., we find relatively lower rates of matrix subject interpretations in those conditions than in 3rd-3rd conditions).

However, the reading time patterns in Experiments 1 and 2 are less clear: In Experiment 1, in the 1st-3rd Condition still showed reading time slowdowns (relative to the baseline 1st-1st condition), which could be taken as an indication that the “blocked” inaccessible matrix subject can still interfere with the local subject. In Experiment 2, the 2nd-3rd Condition showed only marginal reading time slowdowns relative to the 2nd-2nd Condition. Thus, even though the off-line judgments suggest a stable blocking effect, in real-time comprehenders may still briefly consider the “blocked” referents. In Experiment 3, the intervening third person referent seems to exclude the first and second person matrix subject from the initial set of antecedent candidates, as we find no significant reading time slowdowns. Given that Experiments 1 and 2 allow direct speech interpretations, as discussed above, we assume that the results of Experiment 3 are more reliable in this regard.

As a whole, we interpret these results as supporting theoretical claims that blocking is symmetric and third person interveners can also serve as blockers (Tang, 1989; Pollard and Xue, 1998). However, the finding that in Experiment 3, the third person intervener actually exhibited a stronger blocking effect than first or second person interveners, hints that maybe blocking is not fully symmetric. Perhaps first and second person blocking involves a different mechanism than third person based blocking. We leave this as a question for future research.

### Taking a closer look at whether first person pronouns are weaker blockers

Experiment 3 was designed to test (i) whether the first person pronoun and the second person pronoun differ in their effectiveness as blockers, or (ii) whether the high rate of violations of first person blocking in Experiment 1 could be due to comprehenders treating the embedded clauses as quoted direct speech. As described above, a direct speech interpretation would allow “apparent” blocking violations even when the reflexive is actually bound by the local subject. In Experiment 3, we ruled out potential direct speech interpretations by using a verb of hearing (*heard from others* rather than *told others*).

We did not find any evidence in Experiment 3 that first person pronouns are worse blockers than second person pronouns. Interestingly, although both first and second person blocking conditions triggered matrix-subject choices at above-zero rates, the second person blocking condition actually allowed a *higher* rate of matrix subject choices than the first person blocking condition—contrary to the results of Experiments 1 and 2. Thus, after eliminating the possibility of participants representing the embedded clause as direct speech, the first person pronoun seems to create a more stable configuration than the second person pronoun in determining comprehenders' judgments of *ziji*. This finding was unexpected, and merits further investigation.

As a whole, the antecedent choice data in Experiment 3 provide additional evidence for our conclusion that blocking is not a strict, categorical phenomenon. In fact, the first and second person blocking configurations produced *more* matrix subject choices than the two conditions with third person interveners (1st-3rd and 2nd-3rd). However, the reading time data, particularly those from Experiments 2 and 3, show that comprehenders seem to use person feature cues quickly during real-time processing to filter out inaccessible long-distance referents. For example, in Experiment 3, the 3rd-1st Condition had a reading time pattern comparable to that of the 1st-1st Condition at *ziji* and onwards—in other words, we see no signs of a slowdown in the 3rd-1st condition, suggesting that the matrix subject is not competing as a potential antecedent when direct-speech interpretations are ruled out.

Thus, there seems to be a mismatch between comprehenders' on-line performance and off-line antecedent choices: Participants' final responses suggest that, although the local subject is the preferred antecedent for *ziji*, participants still interpret *ziji* as referring to the non-local subject at rates significantly higher than 0. However, at the same time, reading times suggest that when participants process *ziji*, they do not experience slowdowns/competition effects, i.e., reading time patterns suggest that only one antecedent is being considered at the point where *ziji* is processed. This difference between on-line and off-line patterns points to the possibility that the interpretation of *ziji* unfolds over time: it seems that initially, during real-time processing, person-feature cues weigh more heavily and constrain what antecedent candidates get considered. However, participants' off-line interpretations suggest that at some later point, other kinds of information are also integrated and perhaps outweigh the person-feature constraint, resulting in consideration of referents that were initially “blocked” due to the person-feature constraint.

### Implications for models of reference resolution

Our results highlight the role that person features play in guiding the interpretation of reflexives. This contrasts with most existing psycholinguistic models of reflexive processing, which have tended to focus on structural information. For example, Dillon et al. (2009) hypothesize that because *ziji* can be potentially bound by all c-commanding subjects in a discourse, comprehenders should use the c-commanding subjecthood information to search for potential antecedent candidates. Our experiments shed new light on the types of information that guide the interpretation of *ziji*. The finding that comprehenders quickly use person feature cues to guide the search for potential antecedents in real-time suggests that structural information is not the only type of constraint that regulates the real-time processing of *ziji*. In addition, the results from our experiments also suggest that the real-time interpretation of *ziji* can be subtly influenced by comprehenders' mental representations of written texts (i.e., direct vs. indirect speech representations of embedded clauses). These findings are in line with work by Patil et al. (2011), Chen and Vasishth (2011), and Jäger et al. (2015b), who showed that non-structural information also affects the real-time processing of referential forms. The results from Experiments 1–3 also lend support to studies such as Kaiser et al. (2009) that show that comprehenders' antecedent choices do not necessarily follow structural constraints strictly.

## Author contributions

XH and EK conceptualized and designed the experiments. XH collected the data and conducted the statistical analyses. Both XH and EK interpreted the data and wrote the manuscript.

### Conflict of interest statement

The authors declare that the research was conducted in the absence of any commercial or financial relationships that could be construed as a potential conflict of interest.

## Footnotes

^1^It is important to note that alternative approaches to Chomsky's Binding principles have also been proposed. For example, some researchers argue for predicate-based theories, e.g., Reinhart and Reuland (1993).

^2^Example (2a), like our experiments, uses a proper name in the subject position of the embedded clause. This is because a pronoun in that position would allow for coreference between the embedded subject and the matrix subject (e.g., *John*_*i*_ thought that *he*_*i*_ did not like SELF). This would make it impossible to tell whether people interpret *ziji* as referring to the embedded subject or the matrix subject.

^3^It is also worth noting that a situation where first and second person behave differently from third person may also be related to their different positions on the extended animacy hierarchy ((Dixon, 1979), see also (Croft, 2003)), which ranks first and second above third person.

^4^The 11 words are as shown in the pinyin transliteration and the English word-by-word glosses in (4). Some of the words consist of more than one character in Chinese.

^5^In Chinese, there is no construction equivalent to the English structure *hear* + *embedded clause* (e.g., I heard Peter went to Costa Rica). The closest structures are *hear-say* + *embedded clause* and *hear-others-say* + *embedded clause*.
