# Supplementary material for: Processing the Chinese Reflexive “ziji”: Effects of Featural Constraints on Anaphor Resolution
Source: Front Psychol. 2016 Apr 14;7:284. doi: 10.3389/fpsyg.2016.00284 (PMC4830837; doi:10.3389/fpsyg.2016.00284)
Supplement: Supplementary file 2 [file DataSheet2.PDF]

Target items for Experiment 2

Item	Cond	Sentence	Question	Choices
1	1st-1st	你告诉别人你批评自己早上不该把垃圾乱丢。	是谁早上乱丢垃圾？	你 小袁
1	1st-3rd	你告诉别人小慕批评自己早上不该把垃圾乱丢。	是谁早上乱丢垃圾？	你 小慕
1	3rd-1st	小丁告诉别人你批评自己早上不该把垃圾乱丢。	是谁早上乱丢垃圾？	小丁 你
1	3rd-3rd	小丁告诉别人小慕批评自己早上不该把垃圾乱丢。	是谁早上乱丢垃圾？	小丁 小慕
2	1st-1st	你告诉别人你鞭策自己每天坚持认真复习功课。	是谁每天认真复习功课？	你 小杨
2	1st-3rd	你告诉别人小印鞭策自己每天坚持认真复习功课。	是谁每天认真复习功课？	你 小印
2	3rd-1st	小叶告诉别人你鞭策自己每天坚持认真复习功课。	是谁每天认真复习功课？	小叶 你
2	3rd-3rd	小叶告诉别人小印鞭策自己每天坚持认真复习功课。	是谁每天认真复习功课？	小叶 小印
3	1st-1st	你告诉别人你知道自己已经被老板开除了。	是谁被老板开除了？	你 小尤
3	1st-3rd	你告诉别人小包知道自己已经被老板开除了。	是谁被老板开除了？	你 小包
3	3rd-1st	小宁告诉别人你知道自己已经被老板开除了。	是谁被老板开除了？	小宁 你
3	3rd-3rd	小宁告诉别人小包知道自己已经被老板开除了。	是谁被老板开除了？	小宁 小包
4	1st-1st	你告诉别人你责备自己昨天数学考试时作弊。	是谁数学考试作弊？	你 小孟
4	1st-3rd	你告诉别人小冷责备自己昨天数学考试时作弊。	是谁数学考试作弊？	你 小冷
4	1st-1st	小曾告诉别人你责备自己昨天数学考试时作弊。	是谁数学考试作弊？	小曾 你
4	1st-3rd	小曾告诉别人小冷责备自己昨天数学考试时作弊。	是谁数学考试作弊？	小曾 小冷
5	3rd-1st	你告诉别人你相信自己下个学期可以考取大学。	是谁下学期可以考上大学？	你 小奚
5	3rd-3rd	你告诉别人小段相信自己下个学期可以考取大学。	是谁下学期可以考上大学？	你 小段
5	1st-1st	小温告诉别人你相信自己下个学期可以考取大学。	是谁下学期可以考上大学？	小温 你
5	1st-3rd	小温告诉别人小段相信自己下个学期可以考取大学.	是谁下学期可以考上大学？	小温 小段
6	3rd-1st	你告诉别人你督促自己每天晚上认真做作业。	是谁每晚能够认真学习？	你 小吴
6	3rd-3rd	你告诉别人小武督促自己每天晚上认真做作业。	是谁每晚能够认真学习？	你 小武
6	1st-1st	小程告诉别人你督促自己每天晚上认真做作业。	是谁每晚能够认真学习？	小程 你
6	1st-3rd	小程告诉别人小武督促自己每天晚上认真做作业。	是谁每晚能够认真学习？	小程 小武
7	3rd-1st	你告诉别人你吹捧自己非常具备公关社交能力。	谁具有公关社交能力？	你 小卢
7	3rd-3rd	你告诉别人小关吹捧自己非常具备公关社交能力。	谁具有公关社交能力？	你 小关

7	1st-1st	小巩告诉别人你吹捧自己非常具备公关社交能力。	谁具有公关社交能力？	小巩　你
7	1st-3rd	小巩告诉别人小关吹捧自己非常具备公关社交能力。	谁具有公关社交能力？	小巩　小关
8	1st-1st	你告诉别人你清楚自己极度具有摄影的天分。	是谁非常有摄影天分？	你　小元
8	1st-3rd	你告诉别人小乔清楚自己极度具有摄影的天分。	是谁非常有摄影天分？	你　小乔
8	3rd-1st	小商告诉别人你清楚自己极度具有摄影的天分。	是谁非常有摄影天分？	小商　你
8	3rd-3rd	小商告诉别人小乔清楚自己极度具有摄影的天分。	是谁非常有摄影天分？	小商　小乔
9	1st-1st	你告诉别人你预测自己应该可以借到房屋贷款。	谁应该可以借到房屋贷款？	你　小于
9	1st-3rd	你告诉别人小燕预测自己应该可以借到房屋贷款。	谁应该可以借到房屋贷款？	你　小燕
9	3rd-1st	小邢告诉别人你预测自己应该可以借到房屋贷款。	谁应该可以借到房屋贷款？	小邢　你
9	3rd-3rd	小邢告诉别人小燕预测自己应该可以借到房屋贷款。	谁应该可以借到房屋贷款？	小邢　小燕
10	1st-1st	你告诉别人你猜测自己非常有可能会升职。	是谁有可能会被升职？	你　小庄
10	1st-3rd	你告诉别人小左猜测自己非常有可能会升职。	是谁有可能会被升职？	你　小左
10	3rd-1st	小易告诉别人你猜测自己非常有可能会升职。	是谁有可能会被升职？	小易　你
10	3rd-3rd	小易告诉别人小左猜测自己非常有可能会升职。	是谁有可能会被升职？	小易　小左
11	1st-1st	你告诉别人你要求自己每天很早起床锻炼身体。	是谁被要求早起搞锻炼？	你　小商
11	1st-3rd	你告诉别人小石要求自己每天很早起床锻炼身体。	是谁被要求早起搞锻炼？	你　小石
11	1st-1st	小文告诉别人你要求自己每天很早起床锻炼身体。	是谁被要求早起搞锻炼？	小文　你
11	1st-3rd	小文告诉别人小石要求自己每天很早起床锻炼身体。	是谁被要求早起搞锻炼？	小文　小石
12	3rd-1st	你告诉别人你期待自己明年春天考上好大学。	是谁明年可能会考上好大学？	你　小曾
12	3rd-3rd	你告诉别人小惠期待自己明年春天考上好大学。	是谁明年可能会考上好大学？	你　小惠
12	1st-1st	小庄告诉别人你期待自己明年春天考上好大学。	是谁明年可能会考上好大学？	小庄　你
12	1st-3rd	小庄告诉别人小惠期待自己明年春天考上好大学。	是谁明年可能会考上好大学？	小庄　小惠
13	3rd-1st	你告诉别人你希望自己今年暑假可以出国旅游。	是谁想暑假出国旅游？	你　小齐
13	3rd-3rd	你告诉别人小刘希望自己今年暑假可以出国旅游。	是谁想暑假出国旅游？	你　小刘
13	1st-1st	小山告诉别人你希望自己今年暑假可以出国旅游。	是谁想暑假出国旅游？	小山　你
13	1st-3rd	小山告诉别人小刘希望自己今年暑假可以出国旅游。	是谁想暑假出国旅游？	小山　小刘
14	3rd-1st	你告诉别人你强迫自己每天晚上去健身房。	是谁每天晚上去健身房？	你　小魏
14	3rd-3rd	你告诉别人小秋强迫自己每天晚上去健身房。	是谁每天晚上去健身房？	你　小秋
14	1st-1st	小邓告诉别人你强迫自己每天晚上去健身房。	是谁每天晚上去健身房？	小邓　你
14	1st-3rd	小邓告诉别人小秋强迫自己每天晚上去健身房。	是谁每天晚上去健身房？	小邓　小秋

15	1st-1st	你告诉别人你明白自己其实只是一个普通人。	谁只是一个普通人？	你　小韩
15	1st-3rd	你告诉别人小甄明白自己其实只是一个普通人。	谁只是一个普通人？	你　小甄
15	3rd-1st	小赖告诉别人你明白自己其实只是一个普通人。	谁只是一个普通人？	小赖　你
15	3rd-3rd	小赖告诉别人小甄明白自己其实只是一个普通人。	谁只是一个普通人？	小赖　小甄
16	1st-1st	你告诉别人你推荐自己下个星期参加歌唱比赛。	谁要参加歌唱比赛？	你　小陶
16	1st-3rd	你告诉别人小崔推荐自己下个星期参加歌唱比赛。	谁要参加歌唱比赛？	你　小崔
16	3rd-1st	小谷告诉别人你推荐自己下个星期参加歌唱比赛。	谁要参加歌唱比赛？	小谷　你
16	3rd-3rd	小谷告诉别人小崔推荐自己下个星期参加歌唱比赛。	谁要参加歌唱比赛？	小谷　小崔
17	1st-1st	你告诉别人你认为自己期末考试能够拿到高分。	谁可能会期末考试拿满分？	小钟　你
17	1st-3rd	你告诉别人小越认为自己期末考试能够拿到高分。	谁可能会期末考试拿满分？	你　小燕
17	3rd-1st	小聂告诉别人你认为自己期末考试能够拿到高分。	谁可能会期末考试拿满分？	小邢　你
17	3rd-3rd	小聂告诉别人小越认为自己期末考试能够拿到高分。	谁可能会期末考试拿满分？	小邢　小燕
18	1st-1st	你告诉别人你担心自己明年秋天不能按时毕业。	谁有可能明年不能毕业？	小郑　你
18	1st-3rd	你告诉别人小耿担心自己明年秋天不能按时毕业。	谁有可能明年不能毕业？	你　小左
18	1st-1st	小桂告诉别人你担心自己明年秋天不能按时毕业。	谁有可能明年不能毕业？	小易　你
18	1st-3rd	小桂告诉别人小耿担心自己明年秋天不能按时毕业。	谁有可能明年不能毕业？	小易　小左
19	3rd-1st	你告诉别人你觉得自己昨天晚上晚饭吃太多。	是谁昨天晚饭吃太多了？	小赵　你
19	3rd-3rd	你告诉别人小焦觉得自己昨天晚上晚饭吃太多。	是谁昨天晚饭吃太多了？	你　小石
19	1st-1st	小郁告诉别人你觉得自己昨天晚上晚饭吃太多。	是谁昨天晚饭吃太多了？	小文　你
19	1st-3rd	小郁告诉别人小焦觉得自己昨天晚上晚饭吃太多。	是谁昨天晚饭吃太多了？	小文　小石
20	3rd-1st	你告诉别人你痛恨自己不能戒掉吸烟的恶习。	是谁戒不掉抽烟的恶习？	小董　你
20	3rd-3rd	你告诉别人小吉痛恨自己不能戒掉吸烟的恶习。	是谁戒不掉抽烟的恶习？	你　小惠
20	1st-1st	小谭告诉别人你痛恨自己不能戒掉吸烟的恶习。	是谁戒不掉抽烟的恶习？	小庄　你
20	1st-3rd	小谭告诉别人小吉痛恨自己不能戒掉吸烟的恶习。	是谁戒不掉抽烟的恶习？	小庄　小惠
21	3rd-1st	你告诉别人你责怪自己去年夏天不该投资股票	是谁去年夏天投资了股票？	小范　你
21	3rd-3rd	你告诉别人小阳责怪自己去年夏天不该投资股票	是谁去年夏天投资了股票？	你　小刘
21	1st-1st	小柴告诉别人你责怪自己去年夏天不该投资股票	是谁去年夏天投资了股票？	小山　你
21	1st-3rd	小柴告诉别人小阳责怪自己去年夏天不该投资股票	是谁去年夏天投资了股票？	小山　小刘
22	1st-1st	你告诉别人你同意自己能够胜任经理这个职位。	谁能胜任经理这个职位？	小胡　你
22	1st-3rd	你告诉别人小红同意自己能够胜任经理这个职位。	谁能胜任经理这个职位？	你　小秋

22	3rd-1st	小廖告诉别人你同意自己能够胜任经理这个职位。	谁能胜任经理这个职位？	小邓　你
22	3rd-3rd	小廖告诉别人小红同意自己能够胜任经理这个职位。	谁能胜任经理这个职位？	小邓　小秋
23	1st-1st	你告诉别人你安排自己下个月去云南考察。	是谁会下月去云南考察？	小管　你
23	1st-3rd	你告诉别人小佟安排自己下个月去云南考察。	是谁会下月去云南考察？	你　小甄
23	3rd-1st	小楚告诉别人你安排自己下个月去云南考察。	是谁会下月去云南考察？	小赖　你
23	3rd-3rd	小楚告诉别人小佟安排自己下个月去云南考察。	是谁会下月去云南考察？	小赖　小甄
24	1st-1st	你告诉别人你忘记自己今天早上要去医院。	是谁计划早上要去医院的？	小秦　你
24	1st-3rd	你告诉别人小军忘记自己今天早上要去医院。	是谁计划早上要去医院的？	你　小崔
24	3rd-1st	小陆告诉别人你忘记自己今天早上要去医院。	是谁计划早上要去医院的？	小谷　你
24	3rd-3rd	小陆告诉别人小军忘记自己今天早上要去医院。	是谁计划早上要去医院的？	小谷　小崔
25	1st-1st	你告诉别人你推测自己今年年底拿不到分红。	是谁年底可能拿不到分红？	小田　你
25	1st-3rd	你告诉别人小俊推测自己今年年底拿不到分红。	是谁年底可能拿不到分红？	你　小越
25	1st-1st	小黎告诉别人你推测自己今年年底拿不到分红。	是谁年底可能拿不到分红？	小聂　你
25	1st-3rd	小黎告诉别人小俊推测自己今年年底拿不到分红。	是谁年底可能拿不到分红？	小聂　小越
26	3rd-1st	你告诉别人你怀疑自己明年夏天不能按时毕业。	是谁明年夏天可能不能毕业？	小毛　你
26	3rd-3rd	你告诉别人小戈怀疑自己明年夏天不能按时毕业。	是谁明年夏天可能不能毕业？	你　小耿
26	1st-1st	小杭告诉别人你怀疑自己明年夏天不能按时毕业。	是谁明年夏天可能不能毕业？	小桂　你
26	1st-3rd	小杭告诉别人小戈怀疑自己明年夏天不能按时毕业。	是谁明年夏天可能不能毕业？	小桂　小耿
27	3rd-1st	你告诉别人你讨厌自己早上一人去公园散步。	是谁讨厌早上去公园散步？	小沈　你
27	3rd-3rd	你告诉别人小蔡讨厌自己早上一人去公园散步。	是谁讨厌早上去公园散步？	你　小焦
27	1st-1st	小荣告诉别人你讨厌自己早上一人去公园散步。	是谁讨厌早上去公园散步？	小郁　你
27	1st-3rd	小荣告诉别人小蔡讨厌自己早上一人去公园散步。	是谁讨厌早上去公园散步？	小郁　小焦
28	3rd-1st	你告诉别人你估计自己应该可以当选劳动模范。	谁应该可以当选劳模？	小毛　你
28	3rd-3rd	你告诉别人小袁估计自己应该可以当选劳动模范。	谁应该可以当选劳模？	你　小吉
28	1st-1st	小洪告诉别人你估计自己应该可以当选劳动模范。	谁应该可以当选劳模？	小谭　你
28	1st-3rd	小洪告诉别人小袁估计自己应该可以当选劳动模范。	谁应该可以当选劳模？	小谭　小吉
29	1st-1st	你告诉别人你感觉自己今年秋天会找到工作。	谁有可能今年秋天找到工作？	小梁　你
29	1st-3rd	你告诉别人小谢感觉自己今年秋天会找到工作。	谁有可能今年秋天找到工作？	你　小阳
29	3rd-1st	小松告诉别人你感觉自己今年秋天会找到工作。	谁有可能今年秋天找到工作？	小柴　你
29	3rd-3rd	小松告诉别人小谢感觉自己今年秋天会找到工作。	谁有可能今年秋天找到工作？	小柴　小阳

30	1st-1st	你告诉别人你坚信自己明天考试能得到高分。	是谁明天考试可能会拿高分？	小明　你
30	1st-3rd	你告诉别人小贝坚信自己明天考试能得到高分。	是谁明天考试可能会拿高分？	你　小红
30	3rd-1st	小丰告诉别人你坚信自己明天考试能得到高分。	是谁明天考试可能会拿高分？	小廖　你
30	3rd-3rd	小丰告诉别人小贝坚信自己明天考试能得到高分。	是谁明天考试可能会拿高分？	小廖　小红
31	1st-1st	你告诉别人你赞成自己下个学期担任学生主席。	谁会下学期担任学生主席？	小成　你
31	1st-3rd	你告诉别人小成赞成自己下个学期担任学生主席。	谁会下学期担任学生主席？	你　小佟
31	3rd-1st	小东告诉别人你赞成自己下个学期担任学生主席。	谁会下学期担任学生主席？	小楚　你
31	3rd-3rd	小东告诉别人小成赞成自己下个学期担任学生主席。	谁会下学期担任学生主席？	小楚　小佟
32	1st-1st	你告诉别人你谎称自己去年股票赚了很多钱。	是谁去年股票没赚很多钱？	小张　你
32	1st-3rd	你告诉别人小方谎称自己去年股票赚了很多钱。	是谁去年股票没赚很多钱？	你　小军
32	1st-1st	小池告诉别人你谎称自己去年股票赚了很多钱。	是谁去年股票没赚很多钱？	小陆　你
32	1st-3rd	小池告诉别人小方谎称自己去年股票赚了很多钱。	是谁去年股票没赚很多钱？	小陆　小军
