# Supplementary material for: Processing the Chinese Reflexive “ziji”: Effects of Featural Constraints on Anaphor Resolution
Source: Front Psychol. 2016 Apr 14;7:284. doi: 10.3389/fpsyg.2016.00284 (PMC4830837; doi:10.3389/fpsyg.2016.00284)
Supplement: Supplementary file 3 [file DataSheet3.PDF]

Target items for Experiment 3

Item	Condition	Sentences	Question	Choices
1	1st-1st	我 听 别人 说 我 可以 把 自己 的 文章 给 别人 看。	谁的文章可以给别人看？	{我 小商}
1	1st-3rd	我 听 别人 说 小商 可以 把 自己 的 文章 给 别人 看。	谁的文章可以给别人看？	{我 小商}
1	3rd-1st	小王 听 别人 说 我 可以 把 自己 的 文章 给 别人 看。	谁的文章可以给别人看？	{小王 我}
1	2nd-2nd	你 听 别人 说 你 可以 把 自己 的 文章 给 别人 看。	谁的文章可以给别人看？	{你 小商}
1	2nd-3rd	你 听 别人 说 小商 可以 把 自己 的 文章 给 别人 看。	谁的文章可以给别人看？	{你 小商}
1	3rd-2nd	小王 听 别人 说 你 可以 把 自己 的 文章 给 别人 看。	谁的文章可以给别人看？	{小王 你}
2	1st-1st	我 听 别人 说 我 可以 把 自己 的 作业 借给 同学 参照。	谁的作业可以借给同学参考？	{小李 你}
2	1st-3rd	我 听 别人 说 小方 可以 把 自己 的 作业 借给 同学 参照。	谁的作业可以借给同学参考？	{我 小方}
2	3rd-1st	小李 听 别人 说 我 可以 把 自己 的 作业 借给 同学 参照。	谁的作业可以借给同学参考？	{我 小方}
2	2nd-2nd	你 听 别人 说 你 可以 把 自己 的 作业 借给 同学 参照。	谁的作业可以借给同学参考？	{小李 我}
2	2nd-3rd	你 听 别人 说 小方 可以 把 自己 的 作业 借给 同学 参照。	谁的作业可以借给同学参考？	{你 小方}
2	3rd-2nd	小李 听 别人 说 你 可以 把 自己 的 作业 借给 同学 参照。	谁的作业可以借给同学参考？	{你 小方}
3	1st-1st	我 听 别人 说 我 可以 把 自己 的 相片 放在 网路 上。	谁的相片可以放在网路上？	{你 小张}
3	1st-3rd	我 听 别人 说 小张 可以 把 自己 的 相片 放在 网路 上。	谁的相片可以放在网路上？	{小刘 你}
3	3rd-1st	小刘 听 别人 说 我 可以 把 自己 的 相片 放在 网路 上。	谁的相片可以放在网路上？	{我 小张}
3	2nd-2nd	你 听 别人 说 你 可以 把 自己 的 相片 放在 网路 上。	谁的相片可以放在网路上？	{我 小张}
3	2nd-3rd	你 听 别人 说 小张 可以 把 自己 的 相片 放在 网路 上。	谁的相片可以放在网路上？	{小刘 我}
3	3rd-2nd	小刘 听 别人 说 你 可以 把 自己 的 相片 放在 网路 上。	谁的相片可以放在网路上？	{你 小张}
4	1st-1st	我 听 别人 说 我 可以 把 自己 的 复习 材料 给人 抄。	谁的复习材料可以给别人抄？	{你 小吴}
4	1st-3rd	我 听 别人 说 小吴 可以 把 自己 的 复习 材料 给人 抄。	谁的复习材料可以给别人抄？	{你 小吴}
4	3rd-1st	小张 听 别人 说 我 可以 把 自己 的 复习 材料 给人 抄。	谁的复习材料可以给别人抄？	{小张 你}
4	2nd-2nd	你 听 别人 说 你 可以 把 自己 的 复习 材料 给人 抄。	谁的复习材料可以给别人抄？	{我 小吴}
4	2nd-3rd	你 听 别人 说 小吴 可以 把 自己 的 复习 材料 给人 抄。	谁的复习材料可以给别人抄？	{我 小吴}
4	3rd-2nd	小张 听 别人 说 你 可以 把 自己 的 复习 材料 给人 抄。	谁的复习材料可以给别人抄？	{小张 我}
5	1st-1st	我 听 别人 说 我 可以 把 自己 的 饺子 给 别人 吃。	谁的饺子可以给别人吃？	{小郭 我}
5	1st-3rd	我 听 别人 说 小赵 可以 把 自己 的 饺子 给 别人 吃。	谁的饺子可以给别人吃？	{你 小赵}

5	3rd-1st	小郭 听 别人 说 我 可以 把 自己 的 饺子 给 别人 吃。	谁的饺子可以给别人吃？	{你 小赵}
5	2nd-2nd	你 听 别人 说 你 可以 把 自己 的 饺子 给 别人 吃。	谁的饺子可以给别人吃？	{小郭 你}
5	2nd-3rd	你 听 别人 说 小赵 可以 把 自己 的 饺子 给 别人 吃。	谁的饺子可以给别人吃？	{我 小赵}
5	3rd-2nd	小郭 听 别人 说 你 可以 把 自己 的 饺子 给 别人 吃。	谁的饺子可以给别人吃？	{我 小赵}
6	1st-1st	我 听 别人 说 我 可以 把 自己 的 零食 分给 大家 尝。	谁的零食可以给别人尝？	{我 小刘}
6	1st-3rd	我 听 别人 说 小刘 可以 把 自己 的 零食 分给 大家 尝。	谁的零食可以给别人尝？	{小吴 我}
6	3rd-1st	小吴 听 别人 说 我 可以 把 自己 的 零食 分给 大家 尝。	谁的零食可以给别人尝？	{你 小刘}
6	2nd-2nd	你 听 别人 说 你 可以 把 自己 的 零食 分给 大家 尝。	谁的零食可以给别人尝？	{你 小刘}
6	2nd-3rd	你 听 别人 说 小刘 可以 把 自己 的 零食 分给 大家 尝。	谁的零食可以给别人尝？	{小吴 你}
6	3rd-2nd	小吴 听 别人 说 你 可以 把 自己 的 零食 分给 大家 尝。	谁的零食可以给别人尝？	{我 小刘}
7	1st-1st	我 听 别人 说 我 可以 把 自己 的 经验 跟 同学 分享。	谁的经验可以跟同学分享？	{我 小郭}
7	1st-3rd	我 听 别人 说 小郭 可以 把 自己 的 经验 跟 同学 分享。	谁的经验可以跟同学分享？	{我 小郭}
7	3rd-1st	小商 听 别人 说 我 可以 把 自己 的 经验 跟 同学 分享。	谁的经验可以跟同学分享？	{小商 我}
7	2nd-2nd	你 听 别人 说 你 可以 把 自己 的 经验 跟 同学 分享。	谁的经验可以跟同学分享？	{你 小郭}
7	2nd-3rd	你 听 别人 说 小郭 可以 把 自己 的 经验 跟 同学 分享。	谁的经验可以跟同学分享？	{你 小郭}
7	3rd-2nd	小商 听 别人 说 你 可以 把 自己 的 经验 跟 同学 分享。	谁的经验可以跟同学分享？	{小商 你}
8	1st-1st	我 听 别人 说 我 可以 把 自己 的 成绩 透露 给 别人。	谁的成绩可以被透露出去？	{小钱 你}
8	1st-3rd	我 听 别人 说 小肖 可以 把 自己 的 成绩 透露 给 别人。	谁的成绩可以被透露出去？	{我 小肖}
8	3rd-1st	小钱 听 别人 说 我 可以 把 自己 的 成绩 透露 给 别人。	谁的成绩可以被透露出去？	{我 小肖}
8	2nd-2nd	你 听 别人 说 你 可以 把 自己 的 成绩 透露 给 别人。	谁的成绩可以被透露出去？	{小钱 我}
8	2nd-3rd	你 听 别人 说 小肖 可以 把 自己 的 成绩 透露 给 别人。	谁的成绩可以被透露出去？	{你 小肖}
8	3rd-2nd	小钱 听 别人 说 你 可以 把 自己 的 成绩 透露 给 别人。	谁的成绩可以被透露出去？	{你 小肖}
9	1st-1st	我 听 别人 说 我 可以 把 自己 的 考卷 借给 别人 学习。	谁的考卷可以让别人学习？	{你 小李}
9	1st-3rd	我 听 别人 说 小李 可以 把 自己 的 考卷 借给 别人 学习。	谁的考卷可以让别人学习？	{小赵 你}
9	3rd-1st	小赵 听 别人 说 我 可以 把 自己 的 考卷 借给 别人 学习。	谁的考卷可以让别人学习？	{我 小李}
9	2nd-2nd	你 听 别人 说 你 可以 把 自己 的 考卷 借给 别人 学习。	谁的考卷可以让别人学习？	{我 小李}
9	2nd-3rd	你 听 别人 说 小李 可以 把 自己 的 考卷 借给 别人 学习。	谁的考卷可以让别人学习？	{小赵 我}
9	3rd-2nd	小赵 听 别人 说 你 可以 把 自己 的 考卷 借给 别人 学习。	谁的考卷可以让别人学习？	{你 小李}
10	1st-1st	我 听 别人 说 我 可以 把 自己 的 故事 告诉 朋友 们。	谁的故事可以让别人知道？	{你 小钱}
10	1st-3rd	我 听 别人 说 小钱 可以 把 自己 的 故事 告诉 朋友 们。	谁的故事可以让别人知道？	{你 小钱}

10	3rd-1st	小郑 听 别人 说 我 可以 把 自己 的 故事 告诉 朋友 们。	谁的故事可以让别人知道？	{小郑 你}
10	2nd-2nd	你 听 别人 说 你 可以 把 自己 的 故事 告诉 朋友 们。	谁的故事可以让别人知道？	{我 小钱}
10	2nd-3rd	你 听 别人 说 小钱 可以 把 自己 的 故事 告诉 朋友 们。	谁的故事可以让别人知道？	{我 小钱}
10	3rd-2nd	小郑 听 别人 说 你 可以 把 自己 的 故事 告诉 朋友 们。	谁的故事可以让别人知道？	{小郑 我}
11	1st-1st	我 听 别人 说 我 可以 把 自己 的 经历 说给 熟人 听。	谁的经历可以谁让熟人知道？	{小方 我}
11	1st-3rd	我 听 别人 说 小唐 可以 把 自己 的 经历 说给 熟人 听。	谁的经历可以谁让熟人知道？	{你 小唐}
11	3rd-1st	小方 听 别人 说 我 可以 把 自己 的 经历 说给 熟人 听。	谁的经历可以谁让熟人知道？	{你 小唐}
11	2nd-2nd	你 听 别人 说 你 可以 把 自己 的 经历 说给 熟人 听。	谁的经历可以谁让熟人知道？	{小方 你}
11	2nd-3rd	你 听 别人 说 小唐 可以 把 自己 的 经历 说给 熟人 听。	谁的经历可以谁让熟人知道？	{我 小唐}
11	3rd-2nd	小方 听 别人 说 你 可以 把 自己 的 经历 说给 熟人 听。	谁的经历可以谁让熟人知道？	{我 小唐}
12	1st-1st	我 听 别人 说 我 可以 把 自己 的 包裹 带到 邮局 去。	谁的包裹可以被带到邮局去？	{我 小陈}
12	1st-3rd	我 听 别人 说 小陈 可以 把 自己 的 包裹 带到 邮局 去。	谁的包裹可以被带到邮局去？	{小唐 我}
12	3rd-1st	小唐 听 别人 说 我 可以 把 自己 的 包裹 带到 邮局 去。	谁的包裹可以被带到邮局去？	{你 小陈}
12	2nd-2nd	你 听 别人 说 你 可以 把 自己 的 包裹 带到 邮局 去。	谁的包裹可以被带到邮局去？	{你 小陈}
12	2nd-3rd	你 听 别人 说 小陈 可以 把 自己 的 包裹 带到 邮局 去。	谁的包裹可以被带到邮局去？	{小唐 你}
12	3rd-2nd	小唐 听 别人 说 你 可以 把 自己 的 包裹 带到 邮局 去。	谁的包裹可以被带到邮局去？	{我 小陈}
13	1st-1st	我 听 别人 说 我 可以 把 自己 的 杂志 带到 学校 去。	谁的杂志可以被带到学校去？	{我 小王}
13	1st-3rd	我 听 别人 说 小王 可以 把 自己 的 杂志 带到 学校 去。	谁的杂志可以被带到学校去？	{我 小王}
13	3rd-1st	小肖 听 别人 说 我 可以 把 自己 的 杂志 带到 学校 去。	谁的杂志可以被带到学校去？	{小肖 我}
13	2nd-2nd	你 听 别人 说 你 可以 把 自己 的 杂志 带到 学校 去。	谁的杂志可以被带到学校去？	{你 小王}
13	2nd-3rd	你 听 别人 说 小王 可以 把 自己 的 杂志 带到 学校 去。	谁的杂志可以被带到学校去？	{你 小王}
13	3rd-2nd	小肖 听 别人 说 你 可以 把 自己 的 杂志 带到 学校 去。	谁的杂志可以被带到学校去？	{小肖 你}
14	1st-1st	我 听 别人 说 我 可以 把 自己 的 电话 号码 告诉 学生。	谁的电话号码可以让学生知道？	{小陈 你}
14	1st-3rd	我 听 别人 说 小郑 可以 把 自己 的 电话 号码 告诉 学生。	谁的电话号码可以让学生知道？	{我 小郑}
14	3rd-1st	小陈 听 别人 说 我 可以 把 自己 的 电话 号码 告诉 学生。	谁的电话号码可以让学生知道？	{我 小郑}
14	2nd-2nd	你 听 别人 说 你 可以 把 自己 的 电话 号码 告诉 学生。	谁的电话号码可以让学生知道？	{小陈 我}
14	2nd-3rd	你 听 别人 说 小郑 可以 把 自己 的 电话 号码 告诉 学生。	谁的电话号码可以让学生知道？	{你 小郑}
14	3rd-2nd	小陈 听 别人 说 你 可以 把 自己 的 电话 号码 告诉 学生。	谁的电话号码可以让学生知道？	{你 小郑}
15	1st-1st	我 听 别人 说 我 可以 把 自己 的 意见 告诉 上级 领导。	谁的意见可以让领导知道？	{你 小陈}
15	1st-3rd	我 听 别人 说 小陈 可以 把 自己 的 意见 告诉 上级 领导。	谁的意见可以让领导知道？	{小王 你}

15	3rd-1st	小王 听 别人 说 我 可以 把 自己 的 意见 告诉 上级 领导。	谁的意见可以让领导知道？	{我 小陈}
15	2nd-2nd	你 听 别人 说 你 可以 把 自己 的 意见 告诉 上级 领导。	谁的意见可以让领导知道？	{我 小陈}
15	2nd-3rd	你 听 别人 说 小陈 可以 把 自己 的 意见 告诉 上级 领导。	谁的意见可以让领导知道？	{小王 我}
15	3rd-2nd	小王 听 别人 说 你 可以 把 自己 的 意见 告诉 上级 领导。	谁的意见可以让领导知道？	{你 小陈}
16	1st-1st	我 听 别人 说 我 可以 把 自己 的 建议 跟 大家 商讨。	谁的建议可以给大家商讨？	{你 小郑}
16	1st-3rd	我 听 别人 说 小郑 可以 把 自己 的 建议 跟 大家 商讨。	谁的建议可以给大家商讨？	{你 小郑}
16	3rd-1st	小李 听 别人 说 我 可以 把 自己 的 建议 跟 大家 商讨。	谁的建议可以给大家商讨？	{小李 你}
16	2nd-2nd	你 听 别人 说 你 可以 把 自己 的 建议 跟 大家 商讨。	谁的建议可以给大家商讨？	{我 小郑}
16	2nd-3rd	你 听 别人 说 小郑 可以 把 自己 的 建议 跟 大家 商讨。	谁的建议可以给大家商讨？	{我 小郑}
16	3rd-2nd	小李 听 别人 说 你 可以 把 自己 的 建议 跟 大家 商讨。	谁的建议可以给大家商讨？	{小李 我}
17	1st-1st	我 听 别人 说 我 可以 把 自己 的 论文 给 别人 参考。	谁的论文可以让人参考？	{小刘 我}
17	1st-3rd	我 听 别人 说 小郭 可以 把 自己 的 论文 给 别人 参考。	谁的论文可以让人参考？	{你 小郭}
17	3rd-1st	小刘 听 别人 说 我 可以 把 自己 的 论文 给 别人 参考。	谁的论文可以让人参考？	{你 小郭}
17	2nd-2nd	你 听 别人 说 你 可以 把 自己 的 论文 给 别人 参考。	谁的论文可以让人参考？	{小刘 你}
17	2nd-3rd	你 听 别人 说 小郭 可以 把 自己 的 论文 给 别人 参考。	谁的论文可以让人参考？	{我 小郭}
17	3rd-2nd	小刘 听 别人 说 你 可以 把 自己 的 论文 给 别人 参考。	谁的论文可以让人参考？	{我 小郭}
18	1st-1st	我 听 别人 说 我 可以 把 自己 的 想法 跟 他人 说。	谁的想法可以让他人知道？	{我 小吴}
18	1st-3rd	我 听 别人 说 小吴 可以 把 自己 的 想法 跟 他人 说。	谁的想法可以让他人知道？	{小张 我}
18	3rd-1st	小张 听 别人 说 我 可以 把 自己 的 想法 跟 他人 说。	谁的想法可以让他人知道？	{你 小吴}
18	2nd-2nd	你 听 别人 说 你 可以 把 自己 的 想法 跟 他人 说。	谁的想法可以让他人知道？	{你 小吴}
18	2nd-3rd	你 听 别人 说 小吴 可以 把 自己 的 想法 跟 他人 说。	谁的想法可以让他人知道？	{小张 你}
18	3rd-2nd	小张 听 别人 说 你 可以 把 自己 的 想法 跟 他人 说。	谁的想法可以让他人知道？	{我 小吴}
19	1st-1st	我 听 别人 说 我 可以 把 自己 的 故事 与 朋友 分享。	谁的故事可以被分享？	{我 小钱}
19	1st-3rd	我 听 别人 说 小钱 可以 把 自己 的 故事 与 朋友 分享。	谁的故事可以被分享？	{我 小钱}
19	3rd-1st	小郭 听 别人 说 我 可以 把 自己 的 故事 与 朋友 分享。	谁的故事可以被分享？	{小郭 我}
19	2nd-2nd	你 听 别人 说 你 可以 把 自己 的 故事 与 朋友 分享。	谁的故事可以被分享？	{你 小钱}
19	2nd-3rd	你 听 别人 说 小钱 可以 把 自己 的 故事 与 朋友 分享。	谁的故事可以被分享？	{你 小钱}
19	3rd-2nd	小郭 听 别人 说 你 可以 把 自己 的 故事 与 朋友 分享。	谁的故事可以被分享？	{小郭 你}
20	1st-1st	我 听 别人 说 我 可以 把 自己 的 小说 带去 办公 室。	谁的小说可以被带去办公室？	{小吴 你}
20	1st-3rd	我 听 别人 说 小赵 可以 把 自己 的 小说 带去 办公 室。	谁的小说可以被带去办公室？	{我 小赵}

20	3rd-1st	小吴 听 别人 说 我 可以 把 自己 的 小说 带去 办公 室。	谁的小说可以被带去办公室？	{我 小赵}
20	2nd-2nd	你 听 别人 说 你 可以 把 自己 的 小说 带去 办公 室。	谁的小说可以被带去办公室？	{小吴 我}
20	2nd-3rd	你 听 别人 说 小赵 可以 把 自己 的 小说 带去 办公 室。	谁的小说可以被带去办公室？	{你 小赵}
20	3rd-2nd	小吴 听 别人 说 你 可以 把 自己 的 小说 带去 办公 室。	谁的小说可以被带去办公室？	{你 小赵}
21	1st-1st	我 听 别人 说 我 可以 把 自己 的 做菜 诀窍 告诉 大家。	谁的做菜的诀窍可以让大家知道？	{你 小刘}
21	1st-3rd	我 听 别人 说 小刘 可以 把 自己 的 做菜 诀窍 告诉 大家。	谁的做菜的诀窍可以让大家知道？	{小商 你}
21	3rd-1st	小商 听 别人 说 我 可以 把 自己 的 做菜 诀窍 告诉 大家。	谁的做菜的诀窍可以让大家知道？	{我 小刘}
21	2nd-2nd	你 听 别人 说 你 可以 把 自己 的 做菜 诀窍 告诉 大家。	谁的做菜的诀窍可以让大家知道？	{我 小刘}
21	2nd-3rd	你 听 别人 说 小刘 可以 把 自己 的 做菜 诀窍 告诉 大家。	谁的做菜的诀窍可以让大家知道？	{小商 我}
21	3rd-2nd	小商 听 别人 说 你 可以 把 自己 的 做菜 诀窍 告诉 大家。	谁的做菜的诀窍可以让大家知道？	{你 小刘}
22	1st-1st	我 听 别人 说 我 可以 把 自己 的 备课 资料 复印 一下。	谁的备课材料可以被复印？	{小方 你}
22	1st-3rd	我 听 别人 说 小方 可以 把 自己 的 备课 资料 复印 一下。	谁的备课材料可以被复印？	{小方 你}
22	3rd-1st	小钱 听 别人 说 我 可以 把 自己 的 备课 资料 复印 一下。	谁的备课材料可以被复印？	{你 小钱}
22	2nd-2nd	你 听 别人 说 你 可以 把 自己 的 备课 资料 复印 一下。	谁的备课材料可以被复印？	{小方 我}
22	2nd-3rd	你 听 别人 说 小方 可以 把 自己 的 备课 资料 复印 一下。	谁的备课材料可以被复印？	{小方 我}
22	3rd-2nd	小钱 听 别人 说 你 可以 把 自己 的 备课 资料 复印 一下。	谁的备课材料可以被复印？	{我 小钱}
23	1st-1st	我 听 别人 说 我 可以 把 自己 的 旧书 都 捐赠 出去。	谁的旧书可以被全部捐赠出去？	{我 小赵}
23	1st-3rd	我 听 别人 说 小李 可以 把 自己 的 旧书 都 捐赠 出去。	谁的旧书可以被全部捐赠出去？	{小李 你}
23	3rd-1st	小赵 听 别人 说 我 可以 把 自己 的 旧书 都 捐赠 出去。	谁的旧书可以被全部捐赠出去？	{小李 你}
23	2nd-2nd	你 听 别人 说 你 可以 把 自己 的 旧书 都 捐赠 出去。	谁的旧书可以被全部捐赠出去？	{你 小赵}
23	2nd-3rd	你 听 别人 说 小李 可以 把 自己 的 旧书 都 捐赠 出去。	谁的旧书可以被全部捐赠出去？	{小李 我}
23	3rd-2nd	小赵 听 别人 说 你 可以 把 自己 的 旧书 都 捐赠 出去。	谁的旧书可以被全部捐赠出去？	{小李 我}
24	1st-1st	我 听 别人 说 我 可以 把 自己 的 朋友 介绍 给 别人。	谁的朋友可以介绍给别人？	{小王 我}
24	1st-3rd	我 听 别人 说 小王 可以 把 自己 的 朋友 介绍 给 别人。	谁的朋友可以介绍给别人？	{我 小郑}
24	3rd-1st	小郑 听 别人 说 我 可以 把 自己 的 朋友 介绍 给 别人。	谁的朋友可以介绍给别人？	{小王 你}
24	2nd-2nd	你 听 别人 说 你 可以 把 自己 的 朋友 介绍 给 别人。	谁的朋友可以介绍给别人？	{小王 你}
24	2nd-3rd	你 听 别人 说 小王 可以 把 自己 的 朋友 介绍 给 别人。	谁的朋友可以介绍给别人？	{你 小郑}
24	3rd-2nd	小郑 听 别人 说 你 可以 把 自己 的 朋友 介绍 给 别人。	谁的朋友可以介绍给别人？	{小王 我}
25	1st-1st	我 听 别人 说 我 可以 把 自己 的 笑话 登到 微博 上。	谁的笑话可以放在微博上？	{小肖 我}
25	1st-3rd	我 听 别人 说 小肖 可以 把 自己 的 笑话 登到 微博 上。	谁的笑话可以放在微博上？	{小肖 我}

25	3rd-1st	小方 听 别人 说 我 可以 把 自己 的 笑话 登到 微博 上。	谁的笑话可以放在微博上？	{我 小方}
25	2nd-2nd	你 听 别人 说 你 可以 把 自己 的 笑话 登到 微博 上。	谁的笑话可以放在微博上？	{小肖 你}
25	2nd-3rd	你 听 别人 说 小肖 可以 把 自己 的 笑话 登到 微博 上。	谁的笑话可以放在微博上？	{小肖 你}
25	3rd-2nd	小方 听 别人 说 你 可以 把 自己 的 笑话 登到 微博 上。	谁的笑话可以放在微博上？	{你 小方}
26	1st-1st	我 听 别人 说 我 可以 把 自己 的 烦恼 向 领导 汇报。	谁的烦恼可以向上级汇报？	{你 小唐}
26	1st-3rd	我 听 别人 说 小商 可以 把 自己 的 烦恼 向 领导 汇报。	谁的烦恼可以向上级汇报？	{小商 我}
26	3rd-1st	小唐 听 别人 说 我 可以 把 自己 的 烦恼 向 领导 汇报。	谁的烦恼可以向上级汇报？	{小商 我}
26	2nd-2nd	你 听 别人 说 你 可以 把 自己 的 烦恼 向 领导 汇报。	谁的烦恼可以向上级汇报？	{我 小唐}
26	2nd-3rd	你 听 别人 说 小商 可以 把 自己 的 烦恼 向 领导 汇报。	谁的烦恼可以向上级汇报？	{小商 你}
26	3rd-2nd	小唐 听 别人 说 你 可以 把 自己 的 烦恼 向 领导 汇报。	谁的烦恼可以向上级汇报？	{小商 你}
27	1st-1st	我 听 别人 说 我 可以 把 自己 的 笑话 跟 学生 说。	谁的笑话可以跟学生说？	{小张 你}
27	1st-3rd	我 听 别人 说 小张 可以 把 自己 的 笑话 跟 学生 说。	谁的笑话可以跟学生说？	{你 小肖}
27	3rd-1st	小肖 听 别人 说 我 可以 把 自己 的 笑话 跟 学生 说。	谁的笑话可以跟学生说？	{小张 我}
27	2nd-2nd	你 听 别人 说 你 可以 把 自己 的 笑话 跟 学生 说。	谁的笑话可以跟学生说？	{小张 我}
27	2nd-3rd	你 听 别人 说 小张 可以 把 自己 的 笑话 跟 学生 说。	谁的笑话可以跟学生说？	{我 小肖}
27	3rd-2nd	小肖 听 别人 说 你 可以 把 自己 的 笑话 跟 学生 说。	谁的笑话可以跟学生说？	{小张 你}
28	1st-1st	我 听 别人 说 我 可以 把 自己 的 熟人 带去 学校 参观。	谁的熟人可以参观学校？	{小唐 你}
28	1st-3rd	我 听 别人 说 小唐 可以 把 自己 的 熟人 带去 学校 参观。	谁的熟人可以参观学校？	{小唐 你}
28	3rd-1st	小陈 听 别人 说 我 可以 把 自己 的 熟人 带去 学校 参观。	谁的熟人可以参观学校？	{你 小陈}
28	2nd-2nd	你 听 别人 说 你 可以 把 自己 的 熟人 带去 学校 参观。	谁的熟人可以参观学校？	{小唐 我}
28	2nd-3rd	你 听 别人 说 小唐 可以 把 自己 的 熟人 带去 学校 参观。	谁的熟人可以参观学校？	{小唐 我}
28	3rd-2nd	小陈 听 别人 说 你 可以 把 自己 的 熟人 带去 学校 参观。	谁的熟人可以参观学校？	{我 小陈}
29	1st-1st	我 听 别人 说 我 可以 把 自己 的 苦闷 与 老师 报告。	谁的苦闷可以向老师报告？	{我 小王}
29	1st-3rd	我 听 别人 说 小郭 可以 把 自己 的 苦闷 与 老师 报告。	谁的苦闷可以向老师报告？	{小郭 你}
29	3rd-1st	小王 听 别人 说 我 可以 把 自己 的 苦闷 与 老师 报告。	谁的苦闷可以向老师报告？	{小郭 你}
29	2nd-2nd	你 听 别人 说 你 可以 把 自己 的 苦闷 与 老师 报告。	谁的苦闷可以向老师报告？	{你 小王}
29	2nd-3rd	你 听 别人 说 小郭 可以 把 自己 的 苦闷 与 老师 报告。	谁的苦闷可以向老师报告？	{小郭 我}
29	3rd-2nd	小王 听 别人 说 你 可以 把 自己 的 苦闷 与 老师 报告。	谁的苦闷可以向老师报告？	{小郭 我}
30	1st-1st	我 听 别人 说 我 可以 把 自己 的 钢笔 借给 同班 同学。	谁的钢笔可以让同学借？	{小陈 我}
30	1st-3rd	我 听 别人 说 小陈 可以 把 自己 的 钢笔 借给 同班 同学。	谁的钢笔可以让同学借？	{我 小李}

30	3rd-1st	小李 听 别人 说 我 可以 把 自己 的 钢笔 借给 同班 同学。	谁的钢笔可以让同学借？	{小陈 你}
30	2nd-2nd	你 听 别人 说 你 可以 把 自己 的 钢笔 借给 同班 同学。	谁的钢笔可以让同学借？	{小陈 你}
30	2nd-3rd	你 听 别人 说 小陈 可以 把 自己 的 钢笔 借给 同班 同学。	谁的钢笔可以让同学借？	{你 小李}
30	3rd-2nd	小李 听 别人 说 你 可以 把 自己 的 钢笔 借给 同班 同学。	谁的钢笔可以让同学借？	{小陈 我}
31	1st-1st	我 听 别人 说 我 可以 把 自己 的 单车 放在 走廊 里。	谁的单车可以放在走廊里？	{小吴 我}
31	1st-3rd	我 听 别人 说 小吴 可以 把 自己 的 单车 放在 走廊 里。	谁的单车可以放在走廊里？	{小吴 我}
31	3rd-1st	小刘 听 别人 说 我 可以 把 自己 的 单车 放在 走廊 里。	谁的单车可以放在走廊里？	{我 小刘}
31	2nd-2nd	你 听 别人 说 你 可以 把 自己 的 单车 放在 走廊 里。	谁的单车可以放在走廊里？	{小吴 你}
31	2nd-3rd	你 听 别人 说 小吴 可以 把 自己 的 单车 放在 走廊 里。	谁的单车可以放在走廊里？	{小吴 你}
31	3rd-2nd	小刘 听 别人 说 你 可以 把 自己 的 单车 放在 走廊 里。	谁的单车可以放在走廊里？	{你 小刘}
32	1st-1st	我 听 别人 说 我 可以 把 自己 的 成果 跟 同事 说。	谁的成果可以让同事知道？	{你 小张}
32	1st-3rd	我 听 别人 说 小钱 可以 把 自己 的 成果 跟 同事 说。	谁的成果可以让同事知道？	{小钱 我}
32	3rd-1st	小张 听 别人 说 我 可以 把 自己 的 成果 跟 同事 说。	谁的成果可以让同事知道？	{小钱 我}
32	2nd-2nd	你 听 别人 说 你 可以 把 自己 的 成果 跟 同事 说。	谁的成果可以让同事知道？	{我 小张}
32	2nd-3rd	你 听 别人 说 小钱 可以 把 自己 的 成果 跟 同事 说。	谁的成果可以让同事知道？	{小钱 你}
32	3rd-2nd	小张 听 别人 说 你 可以 把 自己 的 成果 跟 同事 说。	谁的成果可以让同事知道？	{小钱 你}
33	1st-1st	我 听 别人 说 我 可以 把 自己 的 衬衫 晾在 宿舍 外面。	谁的衬衫可以晾在宿舍外面？	{小张 你}
33	1st-3rd	我 听 别人 说 小张 可以 把 自己 的 衬衫 晾在 宿舍 外面。	谁的衬衫可以晾在宿舍外面？	{你 小郭}
33	3rd-1st	小郭 听 别人 说 我 可以 把 自己 的 衬衫 晾在 宿舍 外面。	谁的衬衫可以晾在宿舍外面？	{小张 我}
33	2nd-2nd	你 听 别人 说 你 可以 把 自己 的 衬衫 晾在 宿舍 外面。	谁的衬衫可以晾在宿舍外面？	{小张 我}
33	2nd-3rd	你 听 别人 说 小张 可以 把 自己 的 衬衫 晾在 宿舍 外面。	谁的衬衫可以晾在宿舍外面？	{我 小郭}
33	3rd-2nd	小郭 听 别人 说 你 可以 把 自己 的 衬衫 晾在 宿舍 外面。	谁的衬衫可以晾在宿舍外面？	{小张 你}
34	1st-1st	我 听 别人 说 我 可以 把 自己 的 垃圾 放在 大门 外。	谁的垃圾可以放在大门外面？	{小肖 你}
34	1st-3rd	我 听 别人 说 小肖 可以 把 自己 的 垃圾 放在 大门 外。	谁的垃圾可以放在大门外面？	{小肖 你}
34	3rd-1st	小吴 听 别人 说 我 可以 把 自己 的 垃圾 放在 大门 外。	谁的垃圾可以放在大门外面？	{你 小吴}
34	2nd-2nd	你 听 别人 说 你 可以 把 自己 的 垃圾 放在 大门 外。	谁的垃圾可以放在大门外面？	{小肖 我}
34	2nd-3rd	你 听 别人 说 小肖 可以 把 自己 的 垃圾 放在 大门 外。	谁的垃圾可以放在大门外面？	{小肖 我}
34	3rd-2nd	小吴 听 别人 说 你 可以 把 自己 的 垃圾 放在 大门 外。	谁的垃圾可以放在大门外面？	{我 小吴}
35	1st-1st	我 听 别人 说 我 可以 把 自己 的 行李 留在 教室 里。	谁的行李可以放在教室里？	{我 小商}
35	1st-3rd	我 听 别人 说 小赵 可以 把 自己 的 行李 留在 教室 里。	谁的行李可以放在教室里？	{小赵 你}

35	3rd-1st	小商 听 别人 说 我 可以 把 自己 的 行李 留在 教室 里。	谁的行李可以放在教室里？	{小赵 你}
35	2nd-2nd	你 听 别人 说 你 可以 把 自己 的 行李 留在 教室 里。	谁的行李可以放在教室里？	{你 小商}
35	2nd-3rd	你 听 别人 说 小赵 可以 把 自己 的 行李 留在 教室 里。	谁的行李可以放在教室里？	{小赵 我}
35	3rd-2nd	小商 听 别人 说 你 可以 把 自己 的 行李 留在 教室 里。	谁的行李可以放在教室里？	{小赵 我}
36	1st-1st	我 听 别人 说 我 可以 把 自己 的 广告 贴在 学校 门口。	谁的广告可以贴在学校门口？	{小李 我}
36	1st-3rd	我 听 别人 说 小李 可以 把 自己 的 广告 贴在 学校 门口。	谁的广告可以贴在学校门口？	{我 小钱}
36	3rd-1st	小钱 听 别人 说 我 可以 把 自己 的 广告 贴在 学校 门口。	谁的广告可以贴在学校门口？	{小李 你}
36	2nd-2nd	你 听 别人 说 你 可以 把 自己 的 广告 贴在 学校 门口。	谁的广告可以贴在学校门口？	{小李 你}
36	2nd-3rd	你 听 别人 说 小李 可以 把 自己 的 广告 贴在 学校 门口。	谁的广告可以贴在学校门口？	{你 小钱}
36	3rd-2nd	小钱 听 别人 说 你 可以 把 自己 的 广告 贴在 学校 门口。	谁的广告可以贴在学校门口？	{小李 我}
37	1st-1st	我 听 别人 说 我 可以 把 自己 的 汽车 停在 操场 旁边。	谁的汽车可以停在操场旁边？	{小商 我}
37	1st-3rd	我 听 别人 说 小商 可以 把 自己 的 汽车 停在 操场 旁边。	谁的汽车可以停在操场旁边？	{小商 我}
37	3rd-1st	小赵 听 别人 说 我 可以 把 自己 的 汽车 停在 操场 旁边。	谁的汽车可以停在操场旁边？	{我 小赵}
37	2nd-2nd	你 听 别人 说 你 可以 把 自己 的 汽车 停在 操场 旁边。	谁的汽车可以停在操场旁边？	{小商 你}
37	2nd-3rd	你 听 别人 说 小商 可以 把 自己 的 汽车 停在 操场 旁边。	谁的汽车可以停在操场旁边？	{小商 你}
37	3rd-2nd	小赵 听 别人 说 你 可以 把 自己 的 汽车 停在 操场 旁边。	谁的汽车可以停在操场旁边？	{你 小赵}
38	1st-1st	我 听 别人 说 我 可以 把 自己 的 油画 送给 单位 领导。	谁的油画可以送给单位领导？	{你 小郑}
38	1st-3rd	我 听 别人 说 小王 可以 把 自己 的 油画 送给 单位 领导。	谁的油画可以送给单位领导？	{小王 我}
38	3rd-1st	小郑 听 别人 说 我 可以 把 自己 的 油画 送给 单位 领导。	谁的油画可以送给单位领导？	{小王 我}
38	2nd-2nd	你 听 别人 说 你 可以 把 自己 的 油画 送给 单位 领导。	谁的油画可以送给单位领导？	{我 小郑}
38	2nd-3rd	你 听 别人 说 小王 可以 把 自己 的 油画 送给 单位 领导。	谁的油画可以送给单位领导？	{小王 你}
38	3rd-2nd	小郑 听 别人 说 你 可以 把 自己 的 油画 送给 单位 领导。	谁的油画可以送给单位领导？	{小王 你}
39	1st-1st	我 听 别人 说 我 可以 把 自己 的 护照 交给 大使 馆。	谁的护照可以交给大使馆？	{小刘 你}
39	1st-3rd	我 听 别人 说 小刘 可以 把 自己 的 护照 交给 大使 馆。	谁的护照可以交给大使馆？	{你 小方}
39	3rd-1st	小方 听 别人 说 我 可以 把 自己 的 护照 交给 大使 馆。	谁的护照可以交给大使馆？	{小刘 我}
39	2nd-2nd	你 听 别人 说 你 可以 把 自己 的 护照 交给 大使 馆。	谁的护照可以交给大使馆？	{小刘 我}
39	2nd-3rd	你 听 别人 说 小刘 可以 把 自己 的 护照 交给 大使 馆。	谁的护照可以交给大使馆？	{我 小方}
39	3rd-2nd	小方 听 别人 说 你 可以 把 自己 的 护照 交给 大使 馆。	谁的护照可以交给大使馆？	{小刘 你}
40	1st-1st	我 听 别人 说 我 可以 把 自己 的 心事 透露 给 别人。	谁的心事可以让别人知道？	{小方 你}
40	1st-3rd	我 听 别人 说 小方 可以 把 自己 的 心事 透露 给 别人。	谁的心事可以让别人知道？	{小方 你}

40	3rd-1st	小唐 听 别人 说 我 可以 把 自己 的 心事 透露 给 别人。	谁的心事可以让别人知道？	{你 小唐}
40	2nd-2nd	你 听 别人 说 你 可以 把 自己 的 心事 透露 给 别人。	谁的心事可以让别人知道？	{小方 我}
40	2nd-3rd	你 听 别人 说 小方 可以 把 自己 的 心事 透露 给 别人。	谁的心事可以让别人知道？	{小方 我}
40	3rd-2nd	小唐 听 别人 说 你 可以 把 自己 的 心事 透露 给 别人。	谁的心事可以让别人知道？	{我 小唐}
41	1st-1st	我 听 别人 说 我 可以 把 自己 的 作品 寄存 在 学校。	谁的作品可以寄存在学校？	{我 小肖}
41	1st-3rd	我 听 别人 说 小郑 可以 把 自己 的 作品 寄存 在 学校。	谁的作品可以寄存在学校？	{小郑 你}
41	3rd-1st	小肖 听 别人 说 我 可以 把 自己 的 作品 寄存 在 学校。	谁的作品可以寄存在学校？	{小郑 你}
41	2nd-2nd	你 听 别人 说 你 可以 把 自己 的 作品 寄存 在 学校。	谁的作品可以寄存在学校？	{你 小肖}
41	2nd-3rd	你 听 别人 说 小郑 可以 把 自己 的 作品 寄存 在 学校。	谁的作品可以寄存在学校？	{小郑 我}
41	3rd-2nd	小肖 听 别人 说 你 可以 把 自己 的 作品 寄存 在 学校。	谁的作品可以寄存在学校？	{小郑 我}
42	1st-1st	我 听 别人 说 我 可以 把 自己 的 不满 告知 商店 经理。	谁的不满可以告知给商店经理？	{小唐 我}
42	1st-3rd	我 听 别人 说 小唐 可以 把 自己 的 不满 告知 商店 经理。	谁的不满可以告知给商店经理？	{我 小陈}
42	3rd-1st	小陈 听 别人 说 我 可以 把 自己 的 不满 告知 商店 经理。	谁的不满可以告知给商店经理？	{小唐 你}
42	2nd-2nd	你 听 别人 说 你 可以 把 自己 的 不满 告知 商店 经理。	谁的不满可以告知给商店经理？	{小唐 你}
42	2nd-3rd	你 听 别人 说 小唐 可以 把 自己 的 不满 告知 商店 经理。	谁的不满可以告知给商店经理？	{你 小陈}
42	3rd-2nd	小陈 听 别人 说 你 可以 把 自己 的 不满 告知 商店 经理。	谁的不满可以告知给商店经理？	{小唐 我}
